# Local Genomic Instability of the *SpTransformer* Gene Family in the Purple Sea Urchin Inferred from BAC Insert Deletions

**DOI:** 10.3390/genes15020222

**Published:** 2024-02-09

**Authors:** Megan A. Barela Hudgell, Farhana Momtaz, Abiha Jafri, Max A. Alekseyev, L. Courtney Smith

**Affiliations:** 1 Department of Biological Sciences, George Washington University, Washington, DC 20052, USA; mhudgell@unm.edu (M.A.B.H.); momtazf@gwmail.gwu.edu (F.M.);; 2 Department of Mathematics and the Computational Biology Institute, George Washington University, Washington, DC 20052, USA; maxal@gwu.edu

**Keywords:** *Strongylocentrotus purpuratus*, short tandem repeats, tandem duplications, long-read assembly, large DNA deletions

## Abstract

The *SpTransformer* (*SpTrf*) gene family in the purple sea urchin, *Strongylocentrotus purpuratus*, encodes immune response proteins. The genes are clustered, surrounded by short tandem repeats, and some are present in genomic segmental duplications. The genes share regions of sequence and include repeats in the coding exon. This complex structure is consistent with putative local genomic instability. Instability of the *SpTrf* gene cluster was tested by 10 days of growth of *Escherichia coli* harboring bacterial artificial chromosome (BAC) clones of sea urchin genomic DNA with inserts containing *SpTrf* genes. After the growth period, the BAC DNA inserts were analyzed for size and *SpTrf* gene content. Clones with multiple *SpTrf* genes showed a variety of deletions, including loss of one, most, or all genes from the cluster. Alternatively, a BAC insert with a single *SpTrf* gene was stable. BAC insert instability is consistent with variations in the gene family composition among sea urchins, the types of *SpTrf* genes in the family, and a reduction in the gene copy number in single coelomocytes. Based on the sequence variability among *SpTrf* genes within and among sea urchins, local genomic instability of the family may be important for driving sequence diversity in this gene family that would be of benefit to sea urchins in their arms race with marine microbes.

## 1. Introduction

There are many examples of gene copy number expansion into immune response families that function in innate immune systems among organisms. Many examples include cell surface, cytoplasmic, and secreted pathogen recognition receptors that bind microbes and/or their molecules and activate the innate immune response in the presence of possible pathogens or opportunists (e.g., [1,2,3]). The resulting gene families are under selection pressure for genes to be retained or to be deleted from the genome and perhaps to diversify based on pathogens and/or environmental variations. The outcome for the increased size and structure of the gene families results in benefits to the host for a broad recognition of the associated microbial populations and survival in its environment. However, the underlying mechanisms for the initial expansion of a single gene copy into a family is not understood. We have proposed previously that genomic instability is part of the complexity that results in gene expansion [4]. Genomic instability (also referred to as genomic fragility) has been the subject of many studies, a significant number debating its origin. Instability is associated with a high density of genomic sequence repeats [5], which are considered to be rapidly evolving compared to the rest of the genome [6]. These repeats are thought to be generated through processes such as conversion, recombination, slipping misalignments, and single-strand annealing [6,7]. Long regions of repeats can result in secondary structures, such as Z-DNA, bulge loops, hairpin loops, four loop junctions, and G-quadruplexes, that may impair cellular processes (reviewed in [6]). The impairment of cellular processes, including DNA replication and repair, can lead to segmental duplications [6,8,9,10,11], elevated recombination rates [12,13], duplications of tRNA genes [14,15], non-homogeneity of gene distribution [16], and expanded regulatory regions [17,18].

**Figure 1:**
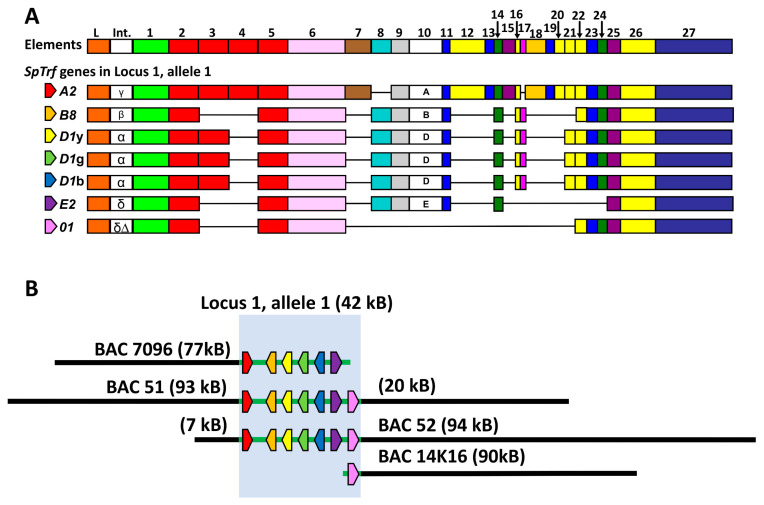
Graphical representation of BAC inserts that contain *SpTrf* gene locus 1, allele 1. (**A**) A graphical alignment of selected *SpTrf* genes showing the element pattern of each gene according to the repeat-based alignment [19]. Genes are composed of two exons; the first encodes the leader (L), and the second encodes the mature protein. All genes have a single, short intron (int) with several identifiable sequences that are indicated by the letters [19]. Exon 2 is a mosaic of elements (all of which are shown with numbers at the top) and is variable among the genes. Element 10 is labeled with different letters that indicate different sequences and define the element pattern. The horizontal black lines indicate missing elements. This figure is modified from Figure 6B in [34]. (**B**) The overlaps among the BAC inserts containing allele 1 from locus 1 are highlighted with the blue box. The black lines to the left and right of the blue box indicate the size of the flanking regions of the inserts that are not part of the gene cluster. This figure is modified from Figure 4A in [35].

The *Transformer* (*Trf*) gene family (previously termed the *185/333* genes) encodes immune response proteins and has been identified in several species of euechinoids [19,20,21,22]. The *SpTrf* genes are present as a family in the purple sea urchin and are upregulated swiftly by phagocytes in adults responding to several different pathogen-associated molecular patterns [23,24,25] and marine bacteria [26,27,28,29,30], and by filipodial cells in the blastocoel of larvae [31]. Several aspects of the structure of the genes and the *SpTrf* family in the purple sea urchin, *Strongylocentrotus purpuratu*s, are consistent with genomic instability. The genes are small, with two exons, of which the second shows significant sequence diversity based on a variety of attributes [19]. It is composed of blocks of similar but non-identical sequences termed elements that are present in mosaic structures that are different among different genes (Figure 1A). Both tandem and interspersed repeats are also present in the second exon. The hypothetical evolutionary history of the tandem repeats suggests local duplications, ectopic duplications and insertions, deletions, and repeat recombination that resulted in two to four imperfect tandem repeats in the extant family [32,33]. In addition to the theoretical recombination among repeats, evidence of possible recombination among genes has also been reported [33]. Although there is significant sequence variability among the genes, they are 88% similar [19]. Consequently, the genes themselves may also be viewed as repeats. The repeats associated with the gene family, along with the sequence similarities among the genes themselves, are consistent with a region of local genomic instability in which evolutionary processes such as recombination may have key functions in sequence diversification and the evolution of both the genes and the family.

The characteristics of the *SpTrf* gene family structure are also consistent with genomic instability. The sequenced family, consisting of 17 alleles, is arranged in the sequenced genome (version 5.0) in two loci [36,37]. The genes are clustered and tightly linked, with intergenic distances ranging from 3.0 kb to 12 kb [32,35,38]. Locus 1 is composed of seven alleles on one chromosome and a mismatch of six alleles on the other (Figure 1B), whereas locus 2 has two genes, each with associated alleles [32]. Short tandem repeats (STRs) composed of two nucleotides, GA, surround each gene, and three regions of GA repeats of 3 kb to 4 kb are present in locus 2 in locations that appear to correspond with the locations of genes in locus 1 [32,35]. A different STR with the repeated sequence of GAT is present in multiple locations within both loci. In addition to the variety of repeats within and surrounding the genes, segmental duplications of 4.3 kb are also present that include duplicated genes, some so recent that their sequences are nearly identical [38], whereas other duplications have quite different sequences [32]. The genome assembly and the variety of BAC inserts that have been sequenced illustrate that the *SpTrf* family loci are riddled with a wide variety of repeats [32,35,37,38].

Genomic instability has been suggested previously, based on the descriptions of the genes and the *SpTrf* gene family structure described above [4]. Quantitative PCR was used to estimate the gene copy numbers in genomes from individual sea urchins, which indicated about 40 to 60 genes per genome [25]. However, the current genome assembly (version 5.0) shows only nine genes in two loci, many of which are annotated incorrectly (Echino-base.org, accessed on 26 October 2023). This underrepresentation has been attributed in part to a computational assembly problem that recognizes the multiple *SpTrf* genes as repeats or alleles and collapses them into single consensus genes, which appear as mixed sequences of two or more alleles or genes [35,38]. Furthermore, the *SpTrf-01* gene identified in a BAC insert sequence and a member of allele 1 in locus 1 [35] is not present in the genome. In an effort to overcome these assembly problems and present a corrected gene family structure, the BAC library that was sequenced for genome assembly (13X coverage of the genome; [39]) was screened for clones harboring *SpTrf* sequences [35]. It was notable that although 75 clones screened positive for *SpTrf* sequences, only 27 BAC clones supported PCR amplification of *SpTrf* sequences after *E. coli* was grown for BAC DNA isolation and analysis. Although this difference might have been the result of a failure of the PCR to amplify many genes for a variety of possible reasons, we propose that an alternative explanation is the instability of the BAC inserts based on the repeats and tight gene clustering that resulted in DNA deletions. This notion is in agreement with the underrepresentation of the *SpTrf* gene copy number in the sequenced genome that was assembled from overlapping BAC insert end sequences [37,40,41]. This hypothesis was tested with repeated inoculation and growth of *E. coli* harboring BAC clones for 10 days. BAC DNA with multiple *SpTrf* genes that was isolated from single colonies after the 10-day growth period had a variety of deletions. Alternatively, the BAC insert with only a single SpTrf gene was stable. Based on these results, we propose that local genomic instability is active in the *SpTrf* gene family in *S. purpuratus* and may be under cellular control to block deletion of the entire gene family. We propose that it is an underlying mechanism for the variability among the genes in the family, which includes sequence diversification that is a benefit for sea urchins in the arms race with marine microbial pathogens.

## 2. Methods

### 2.1. BAC Plasmids, DNA Isolation, and Sequencing

BAC clones were chosen based on their *SpTrf* gene copy numbers (Table 1) [35], which ranged in gene content from a single gene to the full cluster of seven genes in locus 1, allele 1 (Figure 1A). *E. coli* harboring pBACe3.6 plasmids with sea urchin genomic (g)DNA inserts were spread on LB plates with 12.5 µg/mL chloramphenicol (LB/chlor) and grown overnight at 37 °C. Single colonies were inoculated into 5 mL of LB/chlor media and grown overnight at 37 °C with rotation. Bacteria in 1 µL of media were re-inoculated in 5 mL fresh media and grown overnight, which was repeated for 10 days. Bacteria in the final broth culture were spread on LB/chlor plates, single colonies (34–40) were selected per BAC and expanded in LB/chlor media, and BAC plasmid DNA was isolated by the alkaline lysis method as described in [19]. All control BACs (BAC-con) were isolated from *E. coli* harboring the pBACe3.6 plasmids with sea urchin gDNA inserts after a single day of growth as described above. BAC DNA was digested with *Not*I to release the insert from the plasmid and in combination with *Xho*I or *Sac*II, and the inserts were evaluated by pulsed field gel electrophoresis (PFGE; CHEF-DR II Chiller System, Bio-Rad item #1703727) with 1% pulsed field certified agarose (Bio-Rad Laboratories, Hercules, CA, USA) in 0.3X concentration Tris borate EDTA (TBE) gel running buffer (27 mM Tris (pH 7.4), 27 mM boric acid, 0.6 mM EDTA). The PFGE parameters were 6 V/cm, with a switch time of 1–15 s over 16 h [35]. After separation, the gels were soaked in ethidium bromide solution and imaged with a UV imaging system (Gel Logic 1500 Imaging System, Kodak, Rochester, NY, USA). The locations of possible deletions were predicted by virtual restriction enzyme digests (https://nc3.neb.com/NEBcutter; accessed on 18 May 2018; New England Biolabs, Ipswich, MA, USA) based on the full-length insert sequence of each BAC, based on a previous report [42] or as reported here.

The *SpTrf* genes in the BAC inserts were identified by PCR amplicon sizes using primers that amplified all *SpTrf* genes based on annealing sites in the untranslated regions (5′UTR-forward: TAGCATCGGAGAGACCT; 3′UTR-reverse: AAATTCTACACCTCGGCGAC), as described previously [25]. Amplicons were separated by gel electrophoresis and visualized with the Kodak UV imaging system.

### 2.2. BAC Insert Assembly from Sequencing Reads, Alignments, and Dot Plots

BAC DNA (*n* = 3) from single colonies was isolated after 10 days of re-inoculations for clones that showed smaller inserts compared to the full-length original BACs. In addition, BAC-con DNA (*n* = 2) from single colonies was isolated after a single day of growth and showed no insert size differences compared to the full-length original BAC inserts. BACs were grown in LB/chlor media overnight at 37 °C, and the plasmid DNA was isolated by a NucleoBond BAC100 high molecular weight DNA kit (Machery-Nagel Inc., Allentown, PA, USA) and evaluated for *E. coli* genomic DNA contamination by PCR (primers: Ecoli-1F, CGAAGCGACTGGAGCATGTG; Ecoli-1R, ACGCCACATTCGCCAATTC) compared to amplicons of the plasmid (pBACe3.6F, AGCCGTGTAACCGAGCATAGC; pBACe3.6R, GGAACATGACGGTATCTGCGAG). The reaction used PrimeSTAR GXL DNA polymerase (Takara Bio USA, San Jose, CA, USA), and the PCR program was an initial DNA melt at 98 °C for 30 s, 30 cycles of 98 °C for 10 s, 60 °C for 15 s, and 68 °C for 1 min, with a 68 °C extension and 4 °C hold. The amplicons for both pairs of primers were the same size, and inserts from BAC DNA with low levels of contaminating genomic DNA from *E. coli* were sequenced at the University Maryland Institute for Genome Sciences (https://marylandgenomics.org/) using long-read technology (Pacific Biosciences, Menlo Park, CA, USA). BAC insert sequences were assembled using Canu version 2.2 (HiCanu; https://github.com/marbl/canu/releases, accessed on 27 September 2022) [43,44,45] using the following parameters: genome size = 0.03 M, corErrorRate = 0.045, batOptions = “−eg 0.0 −sb 0.001 −dg 3 −dr 0 −ca 2000 −cp 200”, and mhapPipe = false.

Contigs covering each of the BAC inserts that were returned from the HiCanu assembly were aligned by hand in Molecular Evolutionary Genetics Analysis X (MEGAX) against the relevant original BAC insert sequence [35], and a consensus sequence was generated using EMBOSS Cons (https://www.ebi.ac.uk/Tools/msa/emboss_cons/ accessed on 27 September to 15 November 2023) from contigs returned from HiCanu assembly. The consensus sequence was used for further analysis. Sequence assemblies of BAC inserts were verified by BLAST searches of the sequencing reads against the original BAC sequences (GenBank accession numbers KU668451 and KU668452) that have been reported previously [35,38]. Insert sequences for BAC-51-15 and BAC-52-2b are available from GenBank (accession numbers PP082968 and PP082969, respectively). Sequence reads for BAC-42 and BAC-44 are available as raw sequence reads from GenBank (BioSample accession numbers SAMN39322606 and SAMN39322605, respectively).

Dot plots were generated using the YASS genomic similarity search tool (https://bioinfo.univ-lille.fr/yass/index.php, accessed on 3 October 2023) [46] to visualize the deletions in each BAC with a short insert against the original BAC insert sequence. The e-value threshold was set to e^−30^, with the rest of the parameters left at standard settings (scoring matrix: +5, −4, −3, −4; gap costs: −16, −4; X-drop threshold: 30).

Raw PacBio reads for the BAC inserts were mapped against the relevant original BAC insert sequence [35] using minimap2 2.1 with preset parameters [47,48]. The output file was converted from a .sam file to a .bam file, sorted, and indexed using samtools 1.6 [49] before visualization in The Integrative Genomics Viewer (version 2.15.2) [50,51,52,53].

### 2.3. Southern Blots and Riboprobes

BAC clones were digested with *Sal*I and *Not*I, and fragments were separated by gel electrophoresis and transferred to a GeneScreen Plus hybridization membrane (Perkin-Elmer, Waltham, MA, USA) by capillary blotting [35,54]. The filter was evaluated with ^32^P-riboprobes generated with RNA polymerases from linearized gene clones that served as templates to incorporate ^32^P labeled ribonucleotides as described in [23,35,55]. The gene clones chosen for templates had an *A6* element pattern (GenBank accession number EF607716.1), a *B3* element pattern (EF607770.1), and a *D1* element pattern (EF607784.1) [19]. After hybridization with the probes, the filter was exposed to X-ray film at −80 °C, which was processed with developer and fixer, scanned with epi-white light, and imaged with the Kodak Gel Logic 1500 Imaging System.

## 3. Results

### 3.1. Sea Urchin Genomic DNA Harboring the SpTrf Gene Family Is Unstable

**Figure 2:**
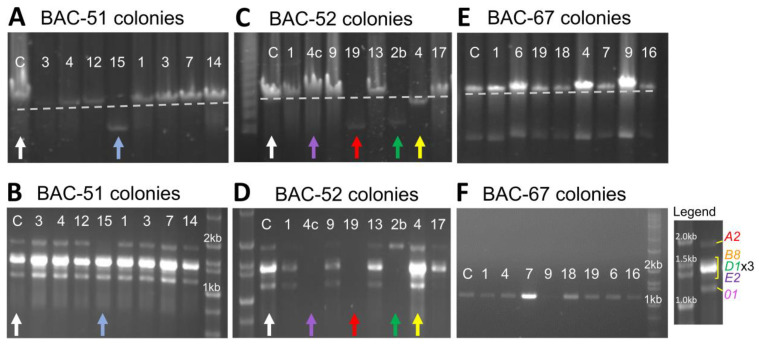
BACs isolated from some colonies after 10 days of growth have small inserts. (**A**,**C**,**E**) Representative *Not*I digests of four BACs containing *SpTrf* genes in locus 1, allele 1 identify BACs with decreased insert sizes. BAC-con clones (C lanes, white arrows) that were grown overnight once show full-length inserts. The C lanes are followed by eight representative samples of BAC DNA isolated from single colonies after 10 days of growth. The colored arrows indicate BACs with small inserts. The first lane in (**C**) shows a PFGE lambda ladder (Bio-Rad Laboratories, Hercules, CA, USA). (**B**,**D**,**F**) PCR amplicons of genes in BAC inserts illustrate the genes in the cluster and identify the genes that are missing after 10 days of growth. Genes were amplified using primers specific for *SpTrf* genes (see Materials and Methods). The colored arrows in (**A**,**C**) correspond to the arrows in (**B**,**D**), respectively. Colored arrows indicate the BACs in which the gene copy number is different from the BAC-con clones (white arrows). The first or last lanes of the gels show the DNA ladder (Hi Lo DNA Standard, Fisher Scientific, Hampton, NH, USA). The legend for *SpTrf* gene amplicons shows the bands that correlate with individual genes based on sizes. *SpTrf*-*A2* is the largest gene; the cluster of bands of intermediate size includes *SpTrf-B8*, -*D1*, and -*E2* (three *D1* genes result in the strongest bands in the center); and *SpTrf*-*01* is the smallest. The marker is the Hi Lo DNA standard.

Genomic instability is predicted for regions of DNA that contain many types of repeats including tightly linked genes with similar sequences [6,56], such as the *SpTrf* genes. An initial characterization of BAC DNA was carried out by *Not*I restriction digests, which released the insert from the pBACe3.6 vector to evaluate size. This approach identified 3% to 10% of the colonies from which BAC DNA was isolated after the 10-day growth period and had inserts that were smaller than expected when they included more than one *SpTrf* gene (Table 1). BAC-51-15 (see Table 1 for the BAC naming conventions) had a small insert compared to BAC-51-con (Figure 2A, blue vs. white arrows), and the corresponding gene amplicons indicated that the *SpTrf-A2* gene was missing (Figure 2B, blue vs. white arrows). The other BACs that originated from BAC-51 all had full-length inserts and a full complement of genes (Figure 2A,B). BAC DNA isolated from *E. coli* colonies that contained BAC-52 showed a variety of changes to the insert sizes (Figure 2C). BAC-52-19, BAC-52-2b, and BAC-52-4 all had small inserts compared to BAC-52-con (Figure 2C, colored arrows) and showed varying numbers of missing *SpTrf* genes (Figure 2D, colored arrows). BAC-52-19 did not support amplification of any *SpTrf* gene, indicating that all were missing (Figure 2D, red arrow). There was a single gene amplicon from BAC-52-2b, which indicated that *SpTrf-A2* was present, but the other genes were missing (Figure 2D, green arrow). BAC-52-4 (Figure 2C,D, yellow arrows), however, showed all gene amplicons, indicating that the full complement of *SpTrf* genes were present (Figure 2D, yellow arrow). Of additional note was BAC-52-4c, which had a full-length insert (Figure 2C, purple arrow), but none of the *SpTrf* genes were amplified (Figure 2D, purple arrow). Al-though the BACs with small inserts showed a variable loss of *SpTrf* genes, the majority of the deletions involved the *SpTrf* gene cluster. In contrast, BAC DNA isolated from multiple colonies that contained BAC-67 with only a single *SpTrf* gene showed no deletions (Table 1, Figure 2E), and the single *SpTrf* gene was maintained (Figure 2F). These results suggested that the repeats within the *SpTrf* cluster were associated with most of the DNA deletions, whereas a single gene separated from the rest of the cluster and with fewer repeats in the insert did not result in DNA deletions.

### 3.2. Deletions to the BAC Inserts Are Positioned in a Variety of Locations

**Figure 3:**
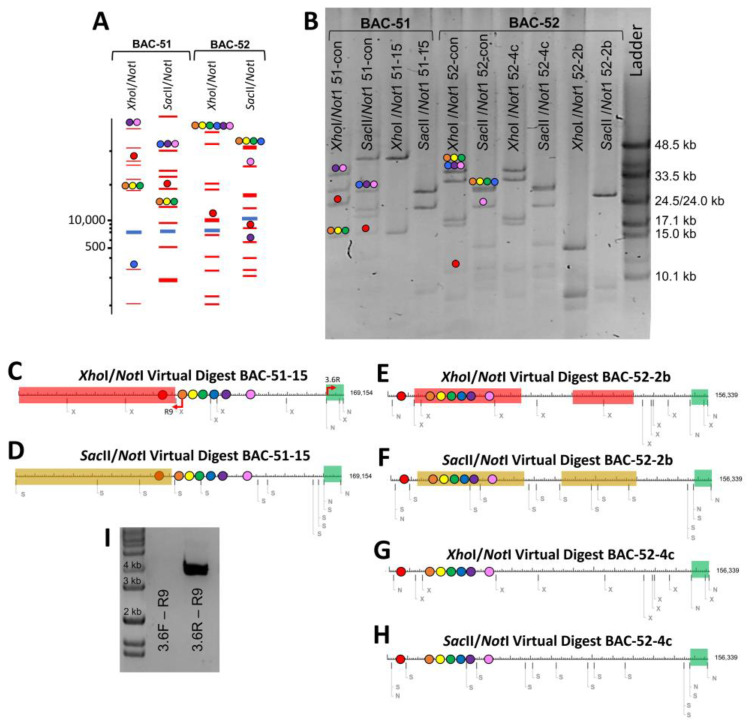
Locations of insert deletions are predicted by restriction digests. (**A**) *Xho*I/*Not*I and *Sac*II/*Not*I virtual digests (https://nc3.neb.com/NEBcutter/ accessed on 18 May 2018) of full-length BAC insert sequences show the fragment sizes and on which fragments the *SpTrf* genes are located. (**B**) *Not*I double digests with *Xho*I or *Sac*II show altered band sizes from BAC clones with small inserts and multiple *SpTrf* genes. BAC-51-con and BAC-52-con with full-length inserts show bands of expected size based on the virtual digests. The ladder is lambda DNA (monocot λ mix; New England Bio-Labs). (**C**–**H**) Maps of virtual digests predict the locations of the deleted regions. The maps are shown in linear format, even though the BAC DNA is circular. The areas in red and yellow indicate the predicted deletions based on results in (**B**). The *SpTrf g*enes are indicated by colored dots based on the gene colors in Figure 1. The green segment indicates the pBACe3.6 vector. Restriction endonuclease sites in the BAC DNA are indicated as N, *Not*I; X, *Xho*I; S, *Sac*II. (**I**) PCR is used to orient and verify the size change in BAC-51-15 (see **C**). Primers (pBACe3.6F or pBACe3.6R) that surround the vector and the R9 primer that is located within each gene (see red arrows in (**C**)) confirm a ~90 kb deletion in BAC-51-15 that results in a 4 kb amplicon. The gel image is edited to delete irrelevant lanes and bring the DNA standard (Hi Lo; Fisher Scientific) next to the lanes of interest.

Although the analysis of BAC insert size and gene copy number indicated a variety of DNA deletions, these results did not identify the locations of the deletions or whether there could be more than one deletion in an individual BAC insert. To address this question, the BAC-con clones and three BAC clones with expected deletions were digested with either *Xho*I/*Not*I or *Sac*II/*Not*I. To predict which fragments corresponded to regions of the BAC inserts to aid in the evaluation of the actual digests, virtual digests were used to evaluate full-length BAC insert sequences for BAC-51 and BAC-52 (Table 1). The virtual double digests generated a wide size distribution of bands and located the seven *SpTrf* genes in specific bands (Figure 3A). While most of the *SpTrf* genes were positioned on the same fragment because of their tight clustering (gene colors in Figure 3A,B are indicated in Figure 1), *SpTrf-A2* and *SpTrf-01* were more likely to be located on different fragments, which correlated with their distant locations at the edges of the cluster. These two genes have larger intergenic regions of 12 kb and 7.3 kb, respectively [35]. However, the *Xho*I/*Not*I digest for BAC-52 showed all genes on the same fragment except for *SpTrf-A2* (Figure 3A). The virtual digest results provided a framework for interpreting the fragments resulting from the actual digests.

The actual digests with *Not*I and either *Xho*I or *Sac*II of the DNA from BAC-51-con, BAC-52-con, and BACs with short inserts (Figure 3B) were compared to the virtual digests (Figure 3A). Differences were used to identify which bands were absent or had changed in size to deduce which regions of the BAC inserts had undergone deletions. Two of the three BACs showed evidence of deletions. The digests of BAC-51-15 indicated a large deletion in which most of the expected bands were missing (Figure 3B). The *Xho*I/*Not*I digest showed a loss of all but one band and a large change in size of another, which the *Sac*II/*Not*I digest showed as a loss of all but three bands. When these deletions and size changes were mapped onto the virtual digest of BAC-51, results predicted a deletion of ~90 kb including the region expected to contain *SpTrf-A2* (Figure 3B–D). This verified the *SpTrf* gene amplicon results for BAC-51-15 in which the *SpTrf-A2* gene was missing (Figure 2D), thereby locating the position of the deleted region within the insert (Figure 3C,D). This deletion was also verified by PCR using the R9 primer (the annealing site is within the coding region of all *SpTrf* genes) and either the pBACe3.6F primer or the pBACe3.6R primer (annealing sites are at the ends of the vector; see red arrows in Figure 3C). An amplicon of 4 kb was generated from BAC-51-15 with pBACe3.6R, which was only feasible if a large deletion had brought the R9 annealing site in *SpTrf-B8* (orange dot) into close proximity to the vector (Figure 3C,I). Results for BAC-52-2b suggested two deletions, of which one removed all genes except for *SpTrf-A2* (Figure 3B,E,F). The second deletion in BAC-52-2b did not alter the *SpTrf* gene cluster; however, it indicated that multiple deletion events could occur in BAC inserts. BAC-52-4c was digested to determine whether any deletions were present given that it showed no change in insert size based on the *NotI* digest compared to the BAC-52-con (Figure 2C, purple arrow) and that it failed to amplify any of the *SpTrf* genes (Figure 2D, purple arrow). Results indicated that there were no differences in the digests for BAC-52-4c and the digests for the BAC-52-con (Figure 3G,H), suggesting that it had no deletions and that the absence of gene amplicons was likely a technical PCR failure (Figure 2D, purple arrow). These findings predicted that inserts that were smaller than expected for some BAC clones were the outcome of deletions. This also suggested that BAC inserts with multiple *SpTrf* genes that are associated with many types of repeats in the gene cluster [35,38] were the basis of the deletions. This was supported by results from BAC-67 with a single *SpTrf* gene and far fewer associated repeats, which did not undergo deletions (Figure 2E,F).

### 3.3. BAC Insert Sequencing and Assembly Verifies Gene Loss and Identifies the Edges of Deletions

**Figure 4:**
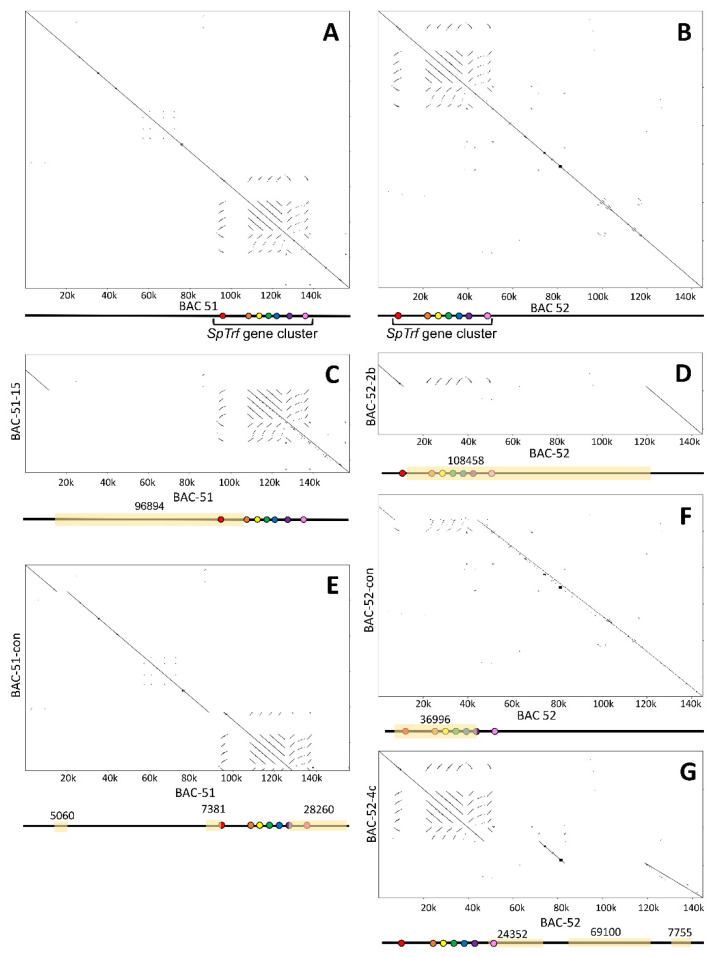
Dot plots of sequenced BAC inserts identify deletions by comparisons to the full-length BAC insert sequences. Dot plots compare each full-length BAC insert to itself and to other BAC inserts that show deletions. The gene cluster maps of BAC-51 or BAC-52 are shown at the bottom of each dot plot with the location of each gene indicated by colored dots (based on the gene colors in Figure 1). Deletions identified by the dot plots are indicated by yellow highlights in the cluster maps and the number of nucleotides in each deletion is indicated. (**A**) BAC-51 vs. self. (**B**) BAC-52 vs. self. (**C**) BAC-51 vs. BAC-51-15. (**D**) BAC-52 vs. BAC-52-2b. (**E**) BAC-52 vs. BAC-52-con. (**F**) BAC-52 vs. BAC-52-con. (**G**) BAC-52 vs. BAC-52-4c.

To verify the results indicating BAC insert deletions based on changes in insert sizes and gene copy numbers, five BACs were sequenced (PacBio) and assembled into full-length insert sequences. BAC-51-15 and BAC-52-2b were selected based on predicted deletions, BAC-52-4c was selected because it maintained the full-length insert after the 10-day growth period but did not appear to have maintained the *SpTrf* genes (Figure 2C,D), and BAC-51-con and BAC-52-con were selected after growth for a single day. Notably, the preparation of large quantities of BAC DNA for sequencing required at least one and often more than one round of growth to acquire enough DNA of sequencing quality. The assembled insert sequences were aligned by hand to either BAC-51 or BAC-52 (Table 1) to verify the locations of the deletions (Supplementary File). Unexpectedly, all sequenced BACs had deletions, including BAC-51-con, BAC-52-con, and BAC-52-4c, which had been identified as full-length without deletions (Figure 2A,C; Table 2). Dot plots were used to illustrate the *SpTrf* gene cluster in BAC-51 and BAC-52 (Figure 4A,B) and to show the locations of deletions in the BACs that underwent the 10-day growth period (Figure 4C–G). The single deletion in BAC-51-15 was consistent with the size and location predicted by the virtual digests and included *SpTrf-A2* (Figure 4C). BAC-52-2b showed one large deletion that removed all of the *SpTrf* genes except for *SpTrf-A2* (Figure 4D) that was not consistent with the virtual digest predictions that suggested two smaller deletions (Figure 3E,F). It appeared that the two predicted deletions may have progressed to a single larger deletion that deleted the region between the two smaller deletions during the preparation for sequencing. Surprisingly, during the preparation of DNA for BAC-51-con, BAC-52-con, and BAC-52-4c for sequencing, these BACs appeared to have also acquired deletions. The insert assembly and dot plot for BAC-51-con showed that, rather than a full-length insert with all *SpTrf* genes in the cluster, three deletions had occurred that truncated *SpTrf-A2* and *SpTrf-E2* and deleted *SpTrf-01* (Figure 4E). Similarly, the dot plot for BAC-52-con compared to BAC-52 also showed a deletion, which truncated *SpTrf-E2* and deleted five genes, although *SpTrf-01* remained (Figure 4F). Furthermore, dot-plot results for BAC-52-4c compared to BAC-52 showed that there were three deletions that removed about 100 kb, including *SpTrf-01*, and the other two deletions were located outside of the gene cluster (Figure 4G). This size change was not evident in the gel image of the *Not*I released insert, which appeared as the same size as BAC-52 (Figure 2C, purple vs. white arrows). The inconsistency within the results required further analysis to identify the source.

**Figure 5:**
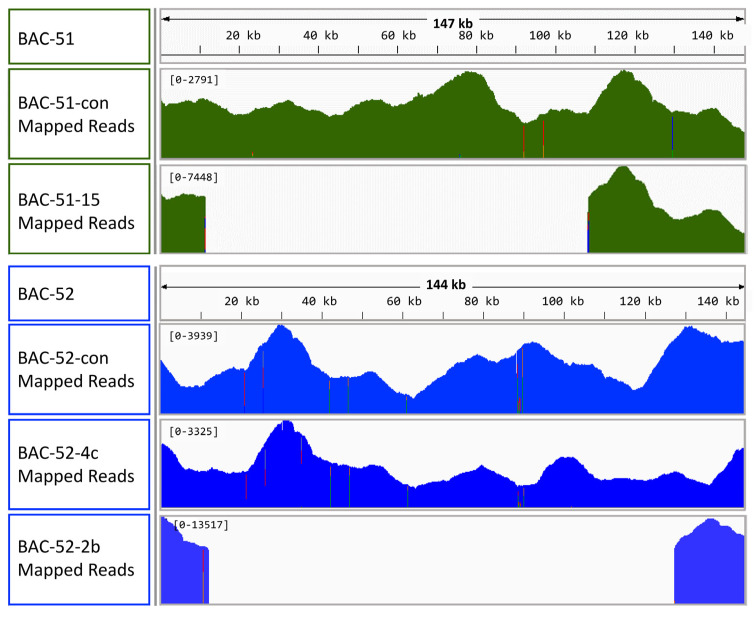
BAC insert assemblies generate false deletions from low-quality sequencing reads. Sequence reads for each BAC are mapped onto reference sequences for BAC-51 (green maps) or BAC-52 (blue maps) that have been reported previously [35]. The lengths of the reference sequences for BAC-51 and BAC-52 are indicated and a ruler is included for every 20 kb. Mapping histograms for each BAC compared to the reference sequence indicate the depth of sequencing coverage and reads per position from the raw PacBio sequencing data. Higher peaks indicate greater coverage, while the absence of peaks indicates no sequence coverage. The colored bars within the histograms indicate nucleotide positions that have increased nucleotide variability across sequence reads. The height of each colored bar indicates the number of sequences for a specific nucleotide. Bar colors indicate nucleotides: red (T), orange (G), light green (A), and dark blue (C).

Because deletions in the full-length BAC inserts did not fit previous analyses of these inserts and because of reported difficulties in assembling sequence reads that include repeats [45,57], as we describe above, verification of the assemblies was required. Raw sequence reads from all five BACs were mapped against the reference sequences submitted to GenBank for BAC-51 and BAC-52 (see Table 1 for accession numbers). Results showed that BAC-51-15 and BAC-51-2b had deletions (Figure 5) in agreement with predictions from gene amplicons and both actual and virtual digests (Figure 2 and Figure 3). BAC-51-con, BAC-52-con, and BAC-52-4c, which were predicted to be full-length with the full complement of genes (Figure 2 and Figure 3), did not show deletions based on the mapping results (Figure 5), which was not in agreement with dot-plot comparisons of the assemblies (Figure 4). The insert assemblies for BAC-51-con, BAC-52-con, and BAC-52-4c were likely the outcome of poor-quality sequence reads that the assembler program omitted when assembling the BAC insert sequences. Consequently, the deletions in these BACs were deemed to be assembly artifacts and illustrated the necessity to employ multiple means to verify sequence assemblies that include repeats. Indeed, the most common reason for low- or poor-quality sequence reads is the presence of highly repetitive stretches of sequences [57], which is the case for these BAC inserts that cover the *SpTrf* gene locus. Furthermore, poor-quality sequences in certain regions of the BAC sequences may be due to variability in deletion presence and location among the individual BAC clones isolated from individual bacterial cells that are present collectively in a single culture. The outcome for sequencing the BAC DNA with inserts that are unstable may be that some of the inserts are full-length and others have random deletions, leading to poor-quality sequence reads at specific locations. Overall, the BAC inserts with predicted and verified deletions showed that one or more *SpTrf* genes were removed.

### 3.4. BAC Deletions Are Flanked by STRs

Many reports of genomic instability and DNA deletions have focused on defects in DNA repair and replication or the locations of deletions and their associations with specific sequences, including STRs, other types of repeats, and poly G sequences that can form G-quadruplexes [58,59]. Consequently, the verified assemblies for BAC-51-15 and BAC-52-2b enabled an investigation of the sequences at the edges of the deletions. The two verified deletions in the BAC inserts identified from sequence alignments were located at or near regions containing STRs or polynucleotides. The first and larger deletion in BAC-51-15 was associated with CT STRs, which spanned 120 base pairs (bp) at the 5′ end of the deletion and a region of CT and TA STRs of over 425 bp at the 3′ end (Table 3; Supplementary File). The second and smaller deletion that was not verified by mapping results and was likely an assembly artifact based on poor-quality sequence reads (Figure 5) was not associated with repetitive sequences (Table 3; Supplementary File). BAC-52-2b had a single deletion, which was bracketed by poly G and poly C (poly G on the complementary strand; see Supplementary File) (Table 3). Poly G stretches presented the possibility of G-quadruplex formation that may impede DNA replication, leading to genomic instability [60]. In general, the locations of the verified deletions in the BAC inserts were associated with STRs and polynucleotides, in agreement with reports of DNA deletion associated with instability hotspots [6,58].

### 3.5. Some BAC Inserts Are Deleted Prior to Analysis

**Figure 6:**
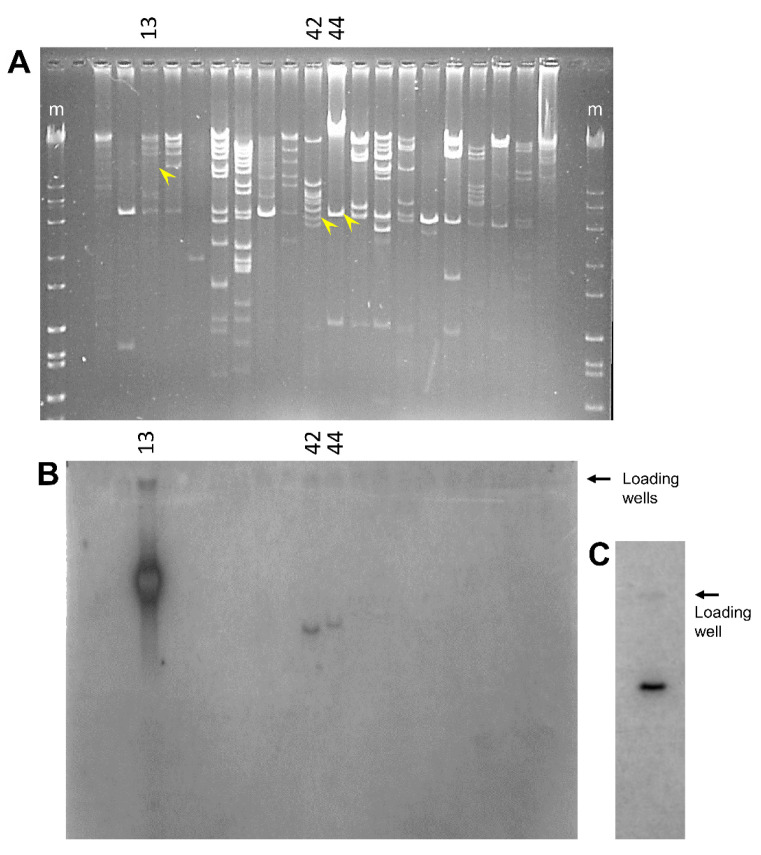
Many BAC inserts do not show *SpTrf* sequences by Southern blot. (**A**) BAC clones that did not amplify *SpTrf* sequences by PCR were digested with *Sal*I and *Not*I, and the fragments were separated by gel electrophoresis, transferred to nylon filters, and evaluated with ^32^P-riboprobes. A subset of those BAC clones are shown. The yellow arrowheads indicate bands with *SpTrf* sequences that correspond to the bands in (**B**). The marker lanes (m) are Hi Lo DNA standards (Fisher Scientific). (**B**) The probes hybridize to three BAC inserts. The raw reads for BAC-42 and BAC-44 are available as BioSamples in GenBank, accession numbers SAMN39322606 and SAMN39322605, respectively. Preliminary sequence analysis of BAC-13 indicates that it includes a region of allele 2 for the *SpTrf* gene cluster in locus 1. Consequently, because this study focused on allele 1 of locus 1, long-read sequencing of the BAC-13 insert was not pursued. (**C**) BAC-7096 with six *SpTrf* genes (see Table 1, Figure 1B) [35,38] is the positive control for the Southern blot.

The initial screen of the BAC library of sea urchin genomic DNA identified 75 BACs with *SpTrf* gene sequences; however, only 27 BAC clones supported PCR amplification of the *SpTrf* genes [35], of which we report results for BAC-51 and BAC-52 above. The clones that did not support amplification of the *SpTrf* genes were evaluated by Southern blots, and BAC-42 and BAC-44 were identified as containing *SpTrf* gene sequences (Figure 6). Based on these conflicting results, these BAC clones were submitted for long-read sequencing. The sequence reads for these BAC inserts could not be assembled into a single sequence and were instead assembled into eight contigs for BAC-44 and three contigs for BAC-42. No *SpTrf* gene sequences were identified within these assemblies, which contradicted the results in the Southern blot. The inconsistencies with BAC-42 and BAC-44 among the other BACs that failed to amplify *SpTrf* sequences suggested that the initial library screens had identified *SpTrf* gene sequences in 75 BAC inserts, but that during the growth and isolation of the BAC DNA for analysis by PFGE and PCR, the inserts underwent deletions. The inference was that these and the other 47 BAC clones were particularly unstable.

## 4. Discussion

### 4.1. Instability of BAC Inserts When Hosted by E. coli

Genomic instability can be lethal when it results in severe genomic fragmentation; however, genomic instability is also a source of potentially beneficial adaptions ([32,61]; reviewed in [62]). For the examples presented here, the BAC inserts are of no benefit to the bacteria; rather, it is the vector which contains the antibiotic resistance that is of benefit to the bacteria under the growth conditions in the presence of chloramphenicol. BAC inserts with repeats are unstable in prokaryotes ([63]; this study), and BACs with smaller inserts benefit *E. coli* because cells with smaller inserts can replicate the BAC DNA more quickly and with lower metabolic cost and therefore may proliferate faster than other cells with larger BAC inserts. The findings presented here suggest that BAC clones with multiple *SpTrf* genes are inherently unstable. Furthermore, deletions within the inserts may progress from multiple small deletions to larger deletions that incorporate the smaller deletions. This was likely the case for BAC-52-2b, with two small deletions identified in early analyses and a single deletion identified in the assembled insert sequence as one large deletion that incorporated the small deletions. Results suggest that the involvement of the STRs and polynucleotides, such as multiple Gs, may promote additional, larger deletions after the initial smaller deletions are initiated. We propose that insert deletions in different BAC clones are initiated at different time points during the 10-day growth period. Perhaps early small deletions are more difficult to detect using our initial methods of restriction digests, whereas later, larger deletions in the same BAC are easier to detect. However, this pattern is not necessarily discernable from our dataset. We propose that the terminal condition of the BAC inserts is the deletion of all *SpTrf* genes, including the associated STRs and other types of repeats, as suggested from the insert sequences of BAC-42 and BAC-44 that do not include *SpTrf* sequences. Once the repeat sequences are deleted, subsequent deletions may cease to occur. Overall, the *SpTrf* gene cluster in BAC inserts appears to be very unstable and tends to undergo deletions. This instability has likely impacted the sequencing phase of the sea urchin genome assembly that employed the BAC library and was one aspect of the poor assembly of *SpTrf* gene loci of only 9 *SpTrf* genes, when 50 to 60 genes have been predicted in the family [33].

### 4.2. Genomic Instability of the SpTrf Gene Family May Underlie Variation in Gene Family Structure and Expression in Sea Urchin Cells

Although our findings suggest that the BAC inserts that include *SpTrf* gene clusters are unstable in *E*. *coli*, the key question is not about the growth benefits of smaller BACs for *E. coli* but whether local genomic instability applies to the *SpTrf* gene loci in the sea urchin genome. If the *SpTrf* gene family is unstable, and given the results presented here suggesting that the genes tend to be deleted from the BAC inserts, how might instability benefit the innate immune response of sea urchins? Genomic instability has been suggested as a source of beneficial genomic variation [62], and the STRs that surround genes and segmental duplications, the repeats within coding regions, and the sequence similarities among genes may all underlie gene duplications and/or deletions and changes in copy number plus subsequent sequence variations among genes [32,38]. STRs and other repeats have been the basis for proposed local genomic instability [4] and a theoretical evolutionary history of the *SpTrf* gene family [32]. Furthermore, there are three regions of GA STRs of several kilobases each that flank the genes in the *SpTrf* locus 2. These STR islands are located in positions that correspond to genes in locus 1, and this has led us to postulate that they may be the remnants of gene deletions that occurred in locus 2 [32,35].

Deletions and local genomic instability for the *SpTrf* gene clusters in the genome is in accord with previous reports on *SpTrf* gene expression and *SpTrf* gene copy numbers in single coelomocytes [29,42]. Immune challenge of sea urchins significantly increases *SpTrf* gene expression [23,24,64], SpTrf protein production [26], and the numbers of SpTrf^+^ coelomocytes in the CF and in other tissues [27,42,65]. Similar results for the *Trf* families have been reported for the sea urchins *Heliocidaris erythrogramma* [20] and *Paracentrotus lividus* [22]. It was assumed that individual phagocytes would express multiple *SpTrf* genes to drive swift responses to invading pathogens. However, when single phagocytes were evaluated for *SpTrf* gene expression, only identical *SpTrf* transcript sequences were identified for individual cells, implying expression of a single *SpTrf* gene per cell [29]. Al-though this result is consistent with gene regulation by promoters and enhancers, there may be an alternative explanation. In an approach to address the question of *SpTrf* gene regulation vs. gene deletion, single small phagocytes were sorted based on the SpTrf proteins on the surface, and red spherule cells that do not express *SpTrf* genes [29] but can be sorted based on the red color of echinochrome, which is characteristic of these cells [42]. Single sperm cells were employed as the control. Because the genes are small enough to be amplified by PCR, variations in amplicon sizes (see the legend in Figure 2) show that the arrays of genes are different among many of the coelomocytes, whereas they are all identical for each sperm for a given animal [42]. Furthermore, the *SpTrf* gene copy number is reduced in most coelomocytes compared to sperm. When the results for *SpTrf* gene expression and gene copy number are integrated, the interpretation suggests that the coelomocytes alter the *SpTrf* gene family, which may restrict and enhance the expression of a single gene per cell. These results are consistent with the notion of local genomic instability of the *SpTrf* gene family and the hypothesis that putative DNA repair mechanisms are employed by the coelomocytes to balance control of locus instability with enhancing *SpTrf* gene sequence diversification. The outcome would be to the advantage of sea urchins in the arms race between their innate immune system and potential pathogens. Parallel results of local genomic instability have been reported for the *agglutinin-like sequence* multi-gene family in *Candida albicans* that contain tandem repeats and encode variations in adhesion proteins for binding to and colonizing host endothelial and epithelial cells, which is an example of a pathogen–host arms race ([66]; reviewed in [62]). Similarly, genes in the fungal *hnwd* family, which functions in allorecognition specificity, encode a series of WD40 repeats that form β propeller structures and show local instability, driving variations in the numbers and sequences of repeats and altering interactions with conspecifics [67]. The killer immunoglobulin-like receptor (*KIR*) locus functions in natural killer cells (reviewed by [68]) and displays gene sequence diversity, tight gene clustering of less than 3 kb of intergenic space, and extraordinarily fast evolutionary change to allelic sequences and the locus structure, as suggested by extensive crossing over [69,70]. The range of repeats in the *KIR* locus is consistent with local genomic instability that drives the varia-bility [71]. The first intron of the *KIR* genes is a minirepeat composed entirely of 30–60 repeats of 19–20 bp [71], and the locus has a number of transposable elements [13], which are repeats that may also result in *KIR* locus instability. Hence, the vertebrate *KIR* and the sea urchin *SpTrf* families both have extensive repeats that may be involved in their respective sequence diversification that may be beneficial in the host-pathogen arms race [72].

Hotspots of genomic instability that are often associated with STRs result in slow or poor DNA replication due to replication fork stalling or reversal, leading to double-strand DNA breaks [73,74]. Non-B DNA structures, such as Z-DNA, hairpins from inverted repeats, and poly G stretches that may form G-quadruplexes can also be the basis for genomic instability at hotspots (reviewed in [75]). G-quadruplexes can block DNA replication fork progression, leading to local genomic instability when helicases fail to unwind G quadruplexes in eukaryotes ([76,77]; reviewed in [78]) and in *E. coli* [79,80]. We report both possibilities for verified DNA deletions in the BAC inserts; CT STRs are associated with the deletion site in BAC-51-15, and poly G regions are associated with the deletion site in BAC-51-2b. In general, the wide variety of DNA repeats that are associated with the *SpTrf* gene family has suggested local genomic instability [32,35,38]. The structural appearance and organization of the family is consistent with instability of the genes located in segmental duplications, STRs that flank both genes and segmental duplications, tandem and interspersed repeats in the coding sequences, sequence variations among the genes, and proposed gene deletions in locus 2.

DNA damage resulting in local genomic instability includes (i) DNA replication stress at fragile sites composed of STRs and other types of repeats [6], (ii) R loops of DNA–RNA hybrids that can lead to double-strand breaks [81,82] and double-strand breaks that occur during general DNA replication (reviewed in [83,84], (iii) highly expressed genomic regions resulting in DNA damage from transcription–replication conflicts [85,86], and (vi) DNA tangles resolved by cleavage followed by incorrect re-ligation [87]. DNA damage and genomic instability are counteracted by DNA repair mechanisms and expression in response to DNA damage in eukaryotes [88]. Genomic instability of the *SpTrf* gene clusters that could result in the deletion of entire loci must be regulated in some way, given that the gene family is maintained in genomes of euechinoid species. Molecular control by coelomocytes to promote, block, or repair chromosomal changes in genomic DNA regions of the *SpTrf* gene clusters may correlate with variations in the level of expression for the DNA repair mechanisms. Hence, there may be a correlation between the expression level of the *SpTrf* genes, which are upregulated significantly in response to immune challenge (reviewed in [89]), and the sea urchin DNA repair mechanisms that may control or regulate local instability of the *SpTrf* gene clusters. Because the *SpTrf* gene family shows an identical composition among single sperm cells from individual sea urchins [42] and given that DNA damage is associated with DNA replication, DNA repair genes may be expected to show elevated expression during mitosis and meiosis in gonads to maintain the structure and membership of the *SpTrf* gene family. Conversely, because individual coelomocytes do not appear to maintain the *SpTrf* gene family equally among cells, DNA repair gene expression may be reduced and/or variable among cells in the axial organ and pharynx where the coelomocytes proliferate [27]. DNA repair genes were identified in initial annotations of the *S. purpuratus* genome sequence (version 2.1) [90], although their expression has not been investigated for the tissues and organs of adult *S. purpuratus*. However, gene expression related to DNA repair has been documented for sea urchin larvae and coelomocytes responding to exposure to genotoxic chemicals [91]. Searches of the *S. purpuratus* genome sequence (version 5.0; www.Echinobase.org; [36], as of 15 December, 2023) result in a number of genes that encode proteins with putative DNA repair functions, including general DNA repair, DNA repair and recombination, excision repair, mismatch repair, double-strand break repair, and DNA cross-link repair, in addition to exonuclease and helicase functions (see also Table S2 in [90]).

Local genomic instability in the *S. purpuratus* genome may not be random, based on the findings from BAC clone insert instability in *E. coli*, yet sea urchin cells must have a means to control or regulate instability to take advantage of the characteristics of the *SpTrf* gene clusters, which are riddled with a wide range of repeats with predicted locations of duplications, deletions, and insertions [32,38,42]. In the case of the *SpTrf* genes in euechinoids, we propose that local genomic instability is an initial and required parameter to drive gene sequence diversification in the population that results in an immune response gene family that keeps pace in the arms race with the marine microbes with which sea urchins share their habitat.

## 5. Conclusions

Genomic instability is driven by the presence of repeats in local regions of the genome. The *SpTrf* gene family, which functions in immune response in the purple sea urchin, is surrounded by multiple types, sizes, and numbers of repetitive sequences, including flanking STRs. We show that the inserts of BAC clones with multiple *SpTrf* genes are unstable in *E. coli* and that with continued growth over time, one or more of the *SpTrf* genes are deleted. The deleted regions are commonly bracketed by STRs or polyG stretches. These results suggest that the repeat-riddled region in the sea urchin genome that includes the *SpTrf* gene family is locally unstable. This may result in expanding, contracting, or maintaining members of the *SpTrf* gene family, which is likely to be beneficial in the arms race against pathogens.

## Figures and Tables

**Table 1:** BACs with multiple *SpTrf* genes show changes in insert sizes and gene copy numbers after a 10-day growth period.

		BAC Insert Screens		SpTrf Gene Copies in BAC Inserts		
BAC Clone Name ^1^	GenBank Accession Number	Colonies Screened for BAC Insert Size	BACs with Deletions	Colonies Screened	Gene Copies Expected	Gene Copies in 8 BACs with Short Inserts
BAC-7096	BK007096	40	4 (10%)	8	3	3, 2, 2, 3, 3, 3, 3, 3
BAC-51	KU668451	34	1 (3%)	8	4	4, 4, 4, 3, 4, 4, 4, 4
BAC-52	KU668452	40	3 (7.5%)	8	4	4, 0, 4, 0, 4, 1, 4, 2
BAC-67	PP082967	40	0 (0%)	8	1	1, 1, 1, 1, 1, 1, 1, 1

^1^ Abbreviated BAC accession numbers are used as clone names.

**Table 2:** The *SpTrf* gene complement is variable among BAC inserts.

BAC Clone Name ^1^	Accession Number	Growth Period (Days)	BAC Insert Size; PFGE ^2^, Sequence	*SpTrf* Genes in Locus 1 Allele 1	Analyses ^3^ to Identify *SpTrf* Gene Complement
BAC-51	KU668451	na ^4^	Full-length 157,542 bp	All genes present	*Not*I digest Gene amplicons
BAC-51-con ^5^	na ^6^	1	Full-length 115,850 bp ^7^	All genes present	*Not*I digest Gene amplicons Virtual digests Insert sequence
BAC-51-15	PP082968	10	Short 67,180 bp ^8^	Deletion of *A2* only	*Not*I digest Gene amplicons Virtual digests Insert sequence
BAC-52	KU668452	na ^4^	Full-length 144,728 bp	All genes present	Gene amplicons
BAC-52-con ^5^	na ^6^	1	Full-length 124,749 bp ^7^	All genes present	*Not*I digest Gene amplicons Virtual digests Insert sequence
BAC-52-4c	na ^6^	10	Full-length 76,739 bp ^7^	All genes deleted ^9^	*Not*I digest Gene amplicons Insert sequence
BAC-52-2b	PP082969	10	Short 36,374bp ^8^	All genes deleted except *A2*	*Not*I digest Gene amplicons Virtual digests Insert sequence
BAC-52-19	na	10	Short Not sequenced	All genes deleted	*Not*I digest Gene amplicons
BAC-52-4	na	10	Short Not sequenced	All genes present	*Not*I digest Gene amplicons Virtual digests

^1^ Abbreviated BAC accession numbers are used as clone names. ^2^ PDFE, pulsed field gel electrophoresis. ^3^ Virtual digests and gene amplicons are shown in Figure 2 and Figure 3. ^4^ na, not applicable. The BAC insert was not sequenced for this study; it was acquired from GenBank. ^5^ con, control. BAC DNA isolated from a single colony grown for a single day served as the control. ^6^ The assembly artifacts precluded submission to GenBank. ^7^ The BAC insert length reported here includes deletion artifacts resulting from the assembly process. ^8^ Insert sequence and deletions are shown in the Supplementary File. ^9^ This is likely a technical PCR failure and a false negative (see Figure 2).

**Table 3:** Regions in BAC insert sequences that are the origins of deletions ^1^.

BAC	Deletion	Local Sequence at Locations of Deletions ^2^
51 vs. 51-15	First, 5′ end	**CTCTCTCTCT**CCCTTT**CTCTCTCTCTCTCTCTCTCTCTCTCTCTCTCTCT**//C**CTCT**AC**CTCTCT**ACTGTAT
	First, 3′ end	**CTCT**CACTTTCTCCTTT**CTCTCTCTCTCTCTCTCTCTCT**//ATCTAT**CTCTATCTCT**ACC
	Second, 5′ end	CT**CACA**GTGTG**AAAA**TGATTTCACA**GTGT**G//ACCAAACTGTGAT**AAA**TAGATTTT**CACAGTGT**G
	Second, 3′ end	TTTACAGTATACCAGAAGTCATTCTTCCCCGATTC//CATCATGCTTTGGAACTTGCTACCTGCTGGA
52 vs. 52-2b	First, 5′ end	**TTTCTTTTTT**GCTCAAC**GGGGGGGGGGG**//TAGTGCATGCGCGGATCCAGGGGAGG**CCCCCCGCCCCCC**AAAA
	First, 3′ end	TACGCAGGA**TTTTTT**CAAGT**GGGGGGGGGGGG**//GGGTTTAACA**TTTTTAAA**TCGGGCCG**AAAATTT**CGCAT

^1^ See the Supplementary File for the full-length sequence alignments. Bold font shows the locations of STRs or polynucleotide sequences. ^2^ //, indicates the location of the deletion. Sequences to the 3ʹ side of the deletion are present in the full-length BAC but are missing in the BACs that contain deletions.

## Data Availability

The sequence data generated in this research are available from GenBank. See Table 1 and Table 2 for accession numbers. The raw sequence reads for BAC-42 and BAC-44 are available from GenBank (BioSample accession numbers SAMN39322606 and SAMN39322605, respectively). The GenBank accession numbers for BAC-51-15 is PP082968, and BAC-52-2b is PP082969. The BAC clones used in this analysis and their genomic DNA library locations are as follows: BAC-51 location is 10B1; BAC-52 location is 4074J14; BAC-67 location is 14K16; BAC-42 location is 4069G2; BAC-44 location is 4069C2, and BAC-13 location is 3020I13. See also: https://www.echinobase.org/echinobase/.

## Supplementary Materials

The following supporting information can be downloaded at: https://www.mdpi.com/article/10.3390/genes15020222/s1. Supplementary File: BAC insert sequence alignments.
