# Supplementary material for: Local Genomic Instability of the SpTransformer Gene Family in the Purple Sea Urchin Inferred from BAC Insert Deletions
Source: Genes (Basel). 2024 Feb 9;15(2):222. doi: 10.3390/genes15020222 (PMC10887614; doi:10.3390/genes15020222)
Supplement: Supplementary file 1 [file genes-15-00222-s001.zip › Barela Hudgell et al revised Supplementary File.pdf]

Supplementary File; BAC insert sequence alignments

# Local genomic instability of the SpTransformer gene family in the purple sea urchin inferred from BAC insert deletions

Megan A. Barela Hudgell, Farhana Momtaz, Abiha Jafri, Max A. Alekseyev, L. Courtney Smith

## Methods

The sequence reads for each BAC insert were assembled into contigs as described in the main paper. The consensus sequences were aligned by hand in Molecular Evolutionary Genetics Analysis X (MEGAX) against the original BAC insert sequences (GenBank Accession numbers KU668451 [BAC-51], KU668452 [BAC-52]) (1). The alignments shown below were generated in BioEdit (version 5.0.9), formatted and exported as rtf files, and imported into this document.

**BAC insert sequence alignments** **Page**

**Alignment 1**. BAC-51 and BAC-51-15 identify the locations of three deletions in BAC-51-15 1
Deletion 1; 96,854 bp
Deletion 2; 2,270 bp*
* likely represents an assembly artifact

**Alignment 2**. BAC-52 and BAC-52-2b identify the locations of one deletion in BAC-52-2b 9
Deletion 1; 108,397 bp

**Alignment 1**. BAC-51 and BAC-51-15

```
                    18910     18920     18930     18940     18950     18960     18970     18980     18990     19000
                ....|....|....|....|....|....|....|....|....|....|....|....|....|....|....|....|....|....|....|....|
BAC-51          TCATTCAACATACCGTAAAATAGCTTCATCATGTTCTTTCAGAAGATTTTAATTTAAAATATTTATTGTGGTTGGGGTATGTTTTCTCCAACTGCAAATG
BAC-51-15       TCATTCAACATACCGTAAAATAGCTTCATCATGTTCTTTCAGAAGATTTTAATTTAAAATATTTATTGTGGTTGGGGTATGTTTTCTCCAACTGCAAATG

                    19010     19020     19030     19040     19050     19060     19070     19080     19090     19100
                ....|....|....|....|....|....|....|....|....|....|....|....|....|....|....|....|....|....|....|....|
BAC-51          ATGTGCATTATATTTTATAAGGTTCATTTTAAATAAGAAACTGTAATAACCTAGTATTTTTGATCAAATATGTTGCTCAGAAGTCTTGAACAGCATTAAT
BAC-51-15       ATGTGCATTATATTTTATAAGGTTCATTTTAAATAAGAAACTGTAATAACCTAGTATTTTTGATCAAATATGTTGCTCAGAAGTCTTGAACAGCATTAAT
```

```
                     19110     19120     19130     19140     19150     19160     19170     19180     19190     19200
                 ....|....|....|....|....|....|....|....|....|....|....|....|....|....|....|....|....|....|....|....|
BAC-51           ACACGACTTAACTACAGACCCTGATTGAATTCCATTTTCAGTCAGCATTGTTGCATAAATTCATTTATTTATGACTTATTTGACTTCTGCATTAAAACAG
BAC-51-15        ACACGACTTAACTACAGACCCTGATTGAATTCCATTTTCAGTCAGCATTGTTGCATAAATTCATTTATTTATGACTTATTTGACTTCTGCATTAAAACAG

                     19210     19220     19230     19240     19250     19260     19270     19280     19290     19300
                 ....|....|....|....|....|....|....|....|....|....|....|....|....|....|....|....|....|....|....|....|
BAC-51           CTTGACAACTTGTTCAGAGGCACCCCCATCGGAAGTGACTTTGTGGGACTAGAGATCACCAGCAGTCGCTTTGATACTAATAGTAACTACGTGATTGGCT
BAC-51-15        CTTGACAACTTGTTCAGAGGCACCCCCATCGGAAGTGACTTTGTGGGACTAGAGATCACCAGCAGTCGCTTTGATACTAATAGTAACTACGTGATTGGCT

                     19310     19320     19330     19340     19350     19360     19370     19380     19390     19400
                 ....|....|....|....|....|....|....|....|....|....|....|....|....|....|....|....|....|....|....|....|
BAC-51           ACTCCATCTACCTGTGTGCTACCTCCCACTACAATGAGGAGAGCATCAAGGATACCTTCAATAGCGCCCTCACAGGGGCTAACATGGACACCTTTGCCGC
BAC-51-15        ACTCCATCTACCTGTGTGCTACCTCCCACTACAATGAGGAGAGCATCAAGGATACCTTCAATAGCGCCCTCACAGGGGCTAACATGGACACCTTTGCCGC

                     19410     19420     19430     19440     19450     19460     19470     19480     19490     19500
                 ....|....|....|....|....|....|....|....|....|....|....|....|....|....|....|....|....|....|....|....|
BAC-51           TTCAAATGTCATTGTGACTGATTCACTTGACTTCAGTAAGTCACTCTACCGAGTTCCAGTTTTTTTAAGGGCAGCAAACATAGACTATCTGTCTAATATT
BAC-51-15        TTCAAATGTCATTGTGACTGATTCACTTGACTTCAGTAAGTCACTCTACCGAGTTCCAGTTTTTTTAAGGGCAGCAAACATAGACTATCTGTCTAATATT

                     19510     19520     19530     19540     19550     19560     19570     19580     19590     19600
                 ....|....|....|....|....|....|....|....|....|....|....|....|....|....|....|....|....|....|....|....|
BAC-51           TTATTGTAATCACTCCATGGCAGTGTTTCGCAAAGACTAAGATCGACTTTATGTTGGACTTAACTATGGCAGGCCATTCATGCCACTGTTTGCTCTCACC
BAC-51-15        TTATTGTAATCACTCCATGGCAGTGTTTCGCAAAGACTAAGATCGACTTTATGTTGGACTTAACTATGGCAGGCCATTCATGCCACTGTTTGCTCTCACC

                     19610     19620     19630     19640     19650     19660     19670     19680     19690     19700
                 ....|....|....|....|....|....|....|....|....|....|....|....|....|....|....|....|....|....|....|....|
BAC-51           ATTTACTGGCATTGGTCTCTCAAGGGACGTACAAATCATGGTCACAAATCTACAGATTTTACATTTTTGGATAATTCATTATTGAAGGAAAAATATTATC
BAC-51-15        ATTTACTGGCATTGGTCTCTCAAGGGACGTACAAATCATGGTCACAAATCTACAGATTTTACATTTTTGGATAATTCATTATTGAAGGAAAAATATTATC

                     19710     19720     19730     19740     19750     19760     19770     19780     19790     19800
                 ....|....|....|....|....|....|....|....|....|....|....|....|....|....|....|....|....|....|....|....|
BAC-51           TAAAGATCATAATGGTGAGAGCAAATAGTGGCATGAACGGCCTGCCATAGTTAAGTCCAACTTAAAGTTGGACTTTCCTCTCTCTCACATGGTGCACTCG
BAC-51-15        TAAAGATCATAATGGTGAGAGCAAATAGTGGCATGAACGGCCTGCCATAGTTAAGTCCAACTTAAAGTTGGACTTTCCTCTCTCTCACATGGTGCACTCG

                     19810     19820     19830     19840     19850     19860     19870     19880     19890     19900
                 ....|....|....|....|....|....|....|....|....|....|....|....|....|....|....|....|....|....|....|....|
BAC-51           CAAGCCTACTGTATAAACTTAAAGTTTATCTTACTCTATGTGAAACACTGCCCATGTCATATTATAATTTGCCTTGTTTGTTGGTGTTCAAATAAACACA
BAC-51-15        CAAGCCTACTGTATAAACTTAAAGTTTATCTTACTCTATGTGAAACACTGCCCATGTCATATTATAATTTGCCTTGTTTGTTGGTGTTCAAATAAACACA

                     19910     19920     19930     19940     19950     19960     19970     19980     19990     20000
                 ....|....|....|....|....|....|....|....|....|....|....|....|....|....|....|....|....|....|....|....|
BAC-51           ATCAGGATGACACAAGTACATGAATTATATATTTCATGGAAACATCATCATTCTTCTTAGCTCAAAGGTAGATAAGGATGCATTCAATAGATCCAGCCAT
BAC-51-15        ATCAGGATGACACAAGTACATGAATTATATATTTCATGGAAACATCATCATTCTTCTTAGCTCAAAGGTAGATAAGGATGCATTCAATAGATCCAGCCAT

                     20010     20020     20030     20040     20050     20060     20070     20080     20090     20100
                 ....|....|....|....|....|....|....|....|....|....|....|....|....|....|....|....|....|....|....|....|
BAC-51           ATAATGAGATCCTCCCAGCACGGTGTTGGTTCATGTATTTGACAGCTATATAATTAACTCATGGTCTCTACTCTCTCTCTCTTTCTCTCTCTTTCTCTCT
BAC-51-15        ATAATGAGATCCTCCCAGCACGGTGTTGGTTCATGTATTTGACAGCTATATAATTAACTCATGGTCTCTACTCTCTCTCTCTTTCTCTCTCTTTCTCTCT
```

```
                      20110     20120     20130     20140     20150     20160     20170     20180     20190     20200
                  ....|....|....|....|....|....|....|....|....|....|....|....|....|....|....|....|....|....|....|....|
BAC-51            CTCTCTTTCTCTCTCTCTCTCTCTCTGTCTCTTTCTCTCTCTCTCCCTTTCTCTCTCTCTCTCTCTCTCTCTCTCTCTCTCCTCTACCTCTCTA
BAC-51-15         CTCTCTTTCTCTCTCTCTCTCTCTCTGTCTCTTTCTCTCTCTCTCCCTTTCTCTCTCTCTCTCTCTCTCTCTCTCTCTCT--------------
```

**Deletion 1, 5' end**

```
                      20210     20220     20230     20240     20250     20260     20270     20280     20290     20300
                  ....|....|....|....|....|....|....|....|....|....|....|....|....|....|....|....|....|....|....|....|
BAC-51            CTGTATGTGTCTGTTTATCTCTCTACAGATGTGGTAGAGTTTAGGGGTCAGTTTACACTGAAGAGCATTGAGAATCCTGGAACCGTTCCTGTCGAATACC
BAC-51-15         ----------------------------------------------------------------------------------------------------

                      20310     20320     20330     20340     20350     20360     20370     20380     20390     20400
                  ....|....|....|....|....|....|....|....|....|....|....|....|....|....|....|....|....|....|....|....|
BAC-51            AATCATGCTGCTCAGATCCATCCAGCAGTACCTACCAAGCACTTCAGAACAGAATCTATGATCATGTAAGTATACCAAGATAGTTATATATAGTCAAATT
BAC-51-15         ----------------------------------------------------------------------------------------------------
```

**Sequences (96,854 bp) in BAC-51 that are deleted in BAC-51-15 are omitted**

```
                      116610    116620    116630    116640    116650    116660    116670    116680    116690    116700
                  ....|....|....|....|....|....|....|....|....|....|....|....|....|....|....|....|....|....|....|....|
BAC-51            GAGAGAGAGAGAGAGATCCTGCTACTACTACTGTCGCTAGATTACTGCTATAAAAACTACTATTTCTTAAAAGGCAAGTACACTCCAAAAATACCTTACT
BAC-51-15         ----------------------------------------------------------------------------------------------------

                      116710    116720    116730    116740    116750    116760    116770    116780    116790    116800
                  ....|....|....|....|....|....|....|....|....|....|....|....|....|....|....|....|....|....|....|....|
BAC-51            TTCAATAGAAATCAGACAATATGTAATCAGACAATTATTTCCCTCACTTTCACTATTATATAAAATTTCTTTTGTCTATCTTCTTCTTCTCTCTCTCCCC
BAC-51-15         ----------------------------------------------------------------------------------------------------

                      116810    116820    116830    116840    116850    116860    116870    116880    116890    116900
                  ....|....|....|....|....|....|....|....|....|....|....|....|....|....|....|....|....|....|....|....|
BAC-51            CCCTCTCTCTCTCTCTCTCTCTCTCTCACACACACACACACAAACGTACACACTCACACACACACTCTCTCTCTCTCCCTCTCTCCATCTCACTCTCATCTC
BAC-51-15         ----------------------------------------------------------------------------------------------------

                      116910    116920    116930    116940    116950    116960    116970    116980    116990    117000
                  ....|....|....|....|....|....|....|....|....|....|....|....|....|....|....|....|....|....|....|....|
BAC-51            TGTCTTTGTCTCTTTGTCTCTCCCTCCCCCCCCCTCTCTCTCTCTCTCTATATATAATCAGACAATATATAATACCTATATCCCTCACTCTCACTTTCTC
BAC-51-15         ----------------------------------------------------------------------------------------------------

                      117010    117020    117030    117040    117050    117060    117070    117080    117090    117100
                  ....|....|....|....|....|....|....|....|....|....|....|....|....|....|....|....|....|....|....|....|
BAC-51            ACTCCCTCTCGTTTTTTTTCTTTCTCTCTCTCTCTCTCTCTCTCTATCTATCTCTATCTCTACCTATCTCTCTCTCTCTCTTTCTTTTTCTCTCTCTCTC
BAC-51-15         ---------------------------------------------ATCTATCTCTATCTCTACCTATCTCTCTCTCTCTCTTTCTTTTTCTCTCTCTCTC
```

**Deletion 1, 3' end**

```
                      117110    117120    117130    117140    117150    117160    117170    117180    117190    117200
                  ....|....|....|....|....|....|....|....|....|....|....|....|....|....|....|....|....|....|....|....|
BAC-51            TCTCTATCAATCTCTATCTCTACCTATCTCTCTCTCTCTCTCTCTCTCTTTCTCTCTCTCTCTCTCTCTCTCTTATCTCAATCGATCAATATCGTGAT
BAC-51-15         TCTCTATCAATCTCTATCTCTACCTATCTCTCTCTCTCTCTCTCTCTCTTTCTCTCTCTCTCTCTCTCTCTCTTATCTCAATCGATCAATATCGTGAT
```

```
                        117210    117220    117230    117240    117250    117260    117270    117280    117290    117300
                    ....|....|....|....|....|....|....|....|....|....|....|....|....|....|....|....|....|....|....|....|
BAC-51              CAATCCAGCTGCATAAGGAAAACAATTATTAGGGGCTATAATATTGAACGCTTCACAAGTATTAGTATGAGTTCAGTTATATATTTTTATTTGACAAAGT
BAC-51-15           CAATCCAGCTGCATAAGGAAAACAATTATTAGGGGCTATAATATTGAACGCTTCACAAGTATTAGTATGAGTTCAGTTATATATTTTTATTTGACAAAGT

                        117310    117320    117330    117340    117350    117360    117370    117380    117390    117400
                    ....|....|....|....|....|....|....|....|....|....|....|....|....|....|....|....|....|....|....|....|
BAC-51              CTTGACAAATTCAGTATCAATAAGAGTTTAACAATTCGTCAACATTTCATGCACCTTTCACTGGCTAAGAGTCTCCTGTATCTGAATTTTAGACACAATA
BAC-51-15           CTTGACAAATTCAGTATCAATAAGAGTTTAACAATTCGTCAACATTTCATGCACCTTTCACTGGCTAAGAGTCTCCTGTATCTGAATTTTAGACACAATA

                        117410    117420    117430    117440    117450    117460    117470    117480    117490    117500
                    ....|....|....|....|....|....|....|....|....|....|....|....|....|....|....|....|....|....|....|....|
BAC-51              ATGTTTTAGTCTCTGGTAAGATATGCATTCTGTCCAAGGAAAGACATTAGTGACTATTAGTATATTTACACAAGCAATCTTTTTTTATACAAATTGTACA
BAC-51-15           ATGTTTTAGTCTCTGGTAAGATATGCATTCTGTCCAAGGAAAGACATTAGTGACTATTAGTATATTTACACAAGCAATCTTTTTTTATACAAATTGTACA

                        117510    117520    117530    117540    117550    117560    117570    117580    117590    117600
                    ....|....|....|....|....|....|....|....|....|....|....|....|....|....|....|....|....|....|....|....|
BAC-51              CCTCGGCGACCACATTGACGTCTTCTTCATTGACTGCGATCTCAACCACTTCAGGTGGTGAAGATGTCGTTACGCTTTCCGTCGTTGGATGCTCCTTCTC
BAC-51-15           CCTCGGCGACCACATTGACGTCTTCTTCATTGACTGCGATCTCAACCACTTCAGGTGGTGAAGATGTCGTTACGCTTTCCGTCGTTGGATGCTCCTTCTC

                        117610    117620    117630    117640    117650    117660    117670    117680    117690    117700
                    ....|....|....|....|....|....|....|....|....|....|....|....|....|....|....|....|....|....|....|....|
BAC-51              CTCACTTTCATTCTCACCCCAACGTCCTCGGTTACCATAGGGATGACGGTGGCCATCACGCCTGGGAGATCCTTCTTCGGTTCTGTTGCGTCTGCCGAAG
BAC-51-15           CTCACTTTCATTCTCACCCCAACGTCCTCGGTTACCATAGGGATGACGGTGGCCATCACGCCTGGGAGATCCTTCTTCGGTTCTGTTGCGTCTGCCGAAG

                        117710    117720    117730    117740    117750    117760    117770    117780    117790    117800
                    ....|....|....|....|....|....|....|....|....|....|....|....|....|....|....|....|....|....|....|....|
BAC-51              GGACGGTCTCCGAAAGGCTTTCTACCGAAGGGGTTGACCCAGAAGGGCCTCATCTCAAACATTGGTCTGTCCTGATCTCCATCTCCGGTCTTGTTGTGAT
BAC-51-15           GGACGGTCTCCGAAAGGCTTTCTACCGAAGGGGTTGACCCAGAAGGGCCTCATCTCAAACATTGGTCTGTCCTGATCTCCATCTCCGGTCTTGTTGTGAT

                        117810    117820    117830    117840    117850    117860    117870    117880    117890    117900
                    ....|....|....|....|....|....|....|....|....|....|....|....|....|....|....|....|....|....|....|....|
BAC-51              GACGATGGGGGTGATCTCCCGTCTCATTATGACCCTGGTGACCTTCTGTGTGGTTGCGTCTGCCGAAGTGGTTGAACCGGAAGGGCCTCGTATCATTTGG
BAC-51-15           GACGATGGGG-TGATCTCCCGTCTCATTATGACCCTGGTGACCTTCTGTGTGGTTGCGTCTGCCGAAGTGGTTGAACCGGAAGGGCCTCGTATCATTTGG

                        117910    117920    117930    117940    117950    117960    117970    117980    117990    118000
                    ....|....|....|....|....|....|....|....|....|....|....|....|....|....|....|....|....|....|....|....|
BAC-51              TTTGTCCTGATCTTGATCTCCCGTCTCATTATGTCCTTGGTGACCCTCTGTCTGGTTATGATGACGGTGGTGGTGGCCTTGGTGGCCATGGTGGCCATGA
BAC-51-15           TTTGTCCTGATCTTGATCTCCCGTCTCATTATGTCCTTGGTGACCCTCTGTCTGGTTATGATGACGGTGGTGGTGGCCTTGGTGGCCATGGTGGCCATGA

                        118010    118020    118030    118040    118050    118060    118070    118080    118090    118100
                    ....|....|....|....|....|....|....|....|....|....|....|....|....|....|....|....|....|....|....|....|
BAC-51              TGGTGAGGGTGAGGACGGCCATCCTCCTCGTTGCGTTCATTGCGCTGACCGAACGGTTGTTCCTCTGGTCGGTCCTGAGGTGGACCTTGACGGTGACCAT
BAC-51-15           TGGTGAGGGTGAGGACGGCCATCCTCCTCGTTGCGTTCATTGCGCTGACCGAACGGTTGTTCCTCTGGTCGGTCCTGAGGTGGACCTTGACGGTGACCAT

                        118110    118120    118130    118140    118150    118160    118170    118180    118190    118200
                    ....|....|....|....|....|....|....|....|....|....|....|....|....|....|....|....|....|....|....|....|
BAC-51              GATGACGACGTCCATGACCATCAAACTGACCGCGCCCTCCTAGACCATCACCAATTTGTTGGGAAGCATCAGTTTCTTCTTCTCCATCACCACGTCTTCC
BAC-51-15           GATGACGACGTCCATGACCATCAAACTGACCGCGCCCTCCTAGACCATCACCAATTTGTTGGGAAGCATCAGTTTCTTCTTCTCCATCACCACGTCTTCC
```

```
                        118210    118220    118230    118240    118250    118260    118270    118280    118290    118300
                ....|....|....|....|....|....|....|....|....|....|....|....|....|....|....|....|....|....|....|....|
BAC-51          TCCTTGGCCGAAGAAAGGTCTTCCTCCAGCACCATCTGGTCTGGAGCCACCAAATCCAGGTCCGTCGAATCTCCTACCACCCATCGGACCGCCATTTTGT
BAC-51-15       TCCTTGGCCGAAGAAAGGTCTTCCTCCAGCACCATCTGGTCTGGAGCCACCAAATCCAGGTCCGTCGAATCTCCTACCACCCATCGGACCGCCATTTTGT

                        118310    118320    118330    118340    118350    118360    118370    118380    118390    118400
                ....|....|....|....|....|....|....|....|....|....|....|....|....|....|....|....|....|....|....|....|
BAC-51          CTGCGTCCATCCATATGTGGGGCACCAAATCCAGGTCCATCGAACCTCCTTCCACCCATTGGTCCACCATCTTGCCTAGATCCACCCATCTGCATTCCAC
BAC-51-15       CTGCGTCCATCCATATGTGGGGCACCAAATCCAGGTCCATCGAACCTCCTTCCACCCATTGGTCCACCATCTTGCCTAGATCCACCCATCTGCATTCCAC

                        118410    118420    118430    118440    118450    118460    118470    118480    118490    118500
                ....|....|....|....|....|....|....|....|....|....|....|....|....|....|....|....|....|....|....|....|
BAC-51          CAGGCCTTCCTCCAAAGCGACCTTGTCCTCTCTCTCTGCCATTCTCATTTCCTCGCCGTTCATTGAAATCTCTTCGTGCGTGAGCTGTAAGGGTTAATGA
BAC-51-15       CAGGCCTTCCTCCAAAGCGACCTTGTCCTCTCTCTCTGCCATTCTCATTTCCTCGCCGTTCATTGAAATCTCTTCGTGCGTGAGCTGTAAGGGTTAATGA

                        118510    118520    118530    118540    118550    118560    118570    118580    118590    118600
                ....|....|....|....|....|....|....|....|....|....|....|....|....|....|....|....|....|....|....|....|
BAC-51          AATAATCGAATTAATTAAGTAATCACATTCTGCATTATTATATTTGGCTCTACATTGGTTTATAGCAGGTGACAAATAAGAATGGTAACTCGCCTTCTTA
BAC-51-15       AATAATCGAATTAATTAAGTAATCACATTCTGCATTATTATATTTGGCTCTACATTGGTTTATAGCAGGTGACAAATAAGAATGGTAACTCGCCTTCTTA

                        118610    118620    118630    118640    118650    118660    118670    118680    118690    118700
                ....|....|....|....|....|....|....|....|....|....|....|....|....|....|....|....|....|....|....|....|
BAC-51          TATTTTT-TCCCTTTATGTTTAATCCTAATCCGTAAGTGCGGTTCGATATTCAAAACGCCTAATTCTGACTGAATTAAATTTGATTACCAGATTGAACGT
BAC-51-15       TATTTTTTTCCCTTTATGTTTAATCCTAATCCGTAAGTGCGGTTCGATATTCAAAACGCCTAATTCTGACTGAATTAAATTTGATTACCAGATTGAACGT

                        118710    118720    118730    118740    118750    118760    118770    118780    118790    118800
                ....|....|....|....|....|....|....|....|....|....|....|....|....|....|....|....|....|....|....|....|
BAC-51          AAACATAATACACATCCTTTGTCATTCAGAAAACGGATTGGTGTAGGCTTAATAAATACAATTAATTATATTGTGTAACGAACAATTTAAAAATGCATAT
BAC-51-15       AAACATAATACACATCCTTTGTCATTCAGAAAACGGATTGGTGTAGGCTTAATAAATACAATTAATTATATTGTGTAACGAACAATTTAAAAATGCATAT

                        118810    118820    118830    118840    118850    118860    118870    118880    118890    118900
                ....|....|....|....|....|....|....|....|....|....|....|....|....|....|....|....|....|....|....|....|
BAC-51          TACAAACTATGAAATAATCATATTTACTTCTGTGAGCCCTTCGTTGGCTTTATATTTAGCACTTATCAAGTAATACCGAGTAATAATTTGATTTCTTACC
BAC-51-15       TACAAACTATGAAATAATCATATTTACTTCTGTGAGCCCTTCGTTGGCTTTATATTTAGCACTTATCAAGTAATACCGAGTAATAATTTGATTTCTTACC

                        118910    118920    118930    118940    118950    118960    118970    118980    118990    119000
                ....|....|....|....|....|....|....|....|....|....|....|....|....|....|....|....|....|....|....|....|
BAC-51          CGAGATAGCAAGAGCAGCCACAATGGCAACGATCAATGTTGCTTTCACCTCCATGTTTGTAAGGTCTCTCCGATGCTACAAGCTTTCTCTAGATTCGTTG
BAC-51-15       CGAGATAGCAAGAGCAGCCACAATGGCAACGATCAATGTTGCTTTCACCTCCATGTTTGTAAGGTCTCTCCGATGCTACAAGCTTTCTCTAGATTCGTTG

                        119010    119020    119030    119040    119050    119060    119070    119080    119090    119100
                ....|....|....|....|....|....|....|....|....|....|....|....|....|....|....|....|....|....|....|....|
BAC-51          CCTTCCAAGAGAGAACTAGCTCCAAACTTAACAACTACCTGCTGGGCCACTGAATTTATAGGTTTTCTACCCTAGATTGATATCTCACACTAGTACCAGA
BAC-51-15       CCTTCCAAGAGAGAACTAGCTCCAAACTTAACAACTACCTGCTGGGCCACTGAATTTATAGGTTTTCTACCCTAGATTGATATCTCACACTAGTACCAGA
```

**Matched sequences (37,500 bp) between the BACs are omitted**

```
                        156610    156620    156630    156640    156650    156660    156670    156680    156690    156700
                ....|....|....|....|....|....|....|....|....|....|....|....|....|....|....|....|....|....|....|....|
BAC-51          GGGCACTTATCAGAGAAGAAAAGGAGTAGTTATAAAAAGGAG---AAGGGGCACTCTGTTTTAATGAAAAGCGCATTTATTAGAAAGGTAAAGGGGCACT
BAC-51-15       GGGCACTTATCAGAGAAGAAAAGGAGTAGTTATAAAAAG-AGAAGAAGGGGCACTCTGTTTTAATGAAAAGCGCATTTATTAGAAAGGTAAAGGGGCACT
```

```
                    156710    156720    156730    156740    156750    156760    156770    156780    156790    156800
            ....|....|....|....|....|....|....|....|....|....|....|....|....|....|....|....|....|....|....|....|
BAC-51      TGTCAAAGTAAAACGAGCATCTCTCATATTTGAATAGGGGCAATTATCGGACGTGAAAGAAGCACTAGTCTTATCTGAAATGAAAAGGAGCAACTTCTTT
BAC-51-15   TGTCAAAGTAAAACGAGCATCTCTCATATTTGAATAGGGGCAATTATCGGACGTGAAAGAAGCACTAGTCTTATCTGAAATGAAAAGGAGCAACTTCTTT

                    156810    156820    156830    156840    156850    156860    156870    156880    156890    156900
            ....|....|....|....|....|....|....|....|....|....|....|....|....|....|....|....|....|....|....|....|
BAC-51      TTACCTGAAAGGGGTCATTCACCAGAGCTGAAAAGAGGCATTTATGAAAAGGAGAAGGGGATCTTTGTTTTAATGGGG-AACTAACCATCAGGAAAAGGG
BAC-51-15   TTACCTGAAAGGGGTCATTCACCAGAGCTGAAAAGAGGC-TTTATGAAAAGGAGAAGGGGATCTTTGTTTTAATGGGGGAACTAACCATCAGGAAAAGGG

                    156910    156920    156930    156940    156950    156960    156970    156980    156990    157000
            ....|....|....|....|....|....|....|....|....|....|....|....|....|....|....|....|....|....|....|....|
BAC-51      GCACCTCTCAAATGTTAATAAGGCAATTATCAGAGGTGAAGGGGGCACTTATTACAAGTGAAAAGGCGCACGTATCGTACCTGAAAAGAGGCACTTACTA
BAC-51-15   GCACCTCTCAAATGTTAATAAGGCAATTATCAGAGGTGAAGGGGGCACTTATTACAAGTGAAAAGGCGCACGTATCGTACCTGAAAAGAGGCACTTACTA

                    157010    157020    157030    157040    157050    157060    157070    157080    157090    157100
            ....|....|....|....|....|....|....|....|....|....|....|....|....|....|....|....|....|....|....|....|
BAC-51      GACATTAAAAGAGGCACACAGCATGAGTAGTAAACACAGCCGTGGAAAAATGTAAGCAACACAAGTTCGTATATAGTCGTACGATGAGCGATTGAAACAA
BAC-51-15   GACATTAAAAGAGGCACACAGCATGAGTAGTAAACACAGCCGTG-AAAAATGTAAGCAACACAAGTTCGTATATAGTCGTACGATGAGCGATTGAAACAA

                    157110    157120    157130    157140    157150    157160    157170    157180    157190    157200
            ....|....|....|....|....|....|....|....|....|....|....|....|....|....|....|....|....|....|....|....|
BAC-51      CGAAGTCTGACGGATCTTTGGTCGAAGAGCACACCTGTCAGCACTGGCGTACTGGTAGGGGGCTAGGGG-AGGGGGGGGGG-CGCCATCCCCCAAATAAA
BAC-51-15   CGAAGTCTGACGGATCTTTGGTCGAAGAGCACACCTGTCAGCACTGGCGTACTGGTAGGGGGCTAGGGGGAGGGGGGGGGGGCGCCATCCCCCAAATAAA

                    157210    157220    157230    157240    157250    157260    157270    157280    157290    157300
            ....|....|....|....|....|....|....|....|....|....|....|....|....|....|....|....|....|....|....|....|
BAC-51      ATTGAACCATGACATATTTTCAATTCCATATACAAGTGATGTATAAATGTTCGTGATATCACATGATTTTCAACAAAATGCGTTTTTCTCCATTTCTGTT
BAC-51-15   ATTGAACCATGACATATTTTCAATTCCATATACAAGTGATGTATAAATGTTCGTGATATCACATGATTTTCAACAAAATGCGTTTTTCTCCATTTCTGTT

                    157310    157320    157330    157340    157350    157360    157370    157380    157390    157400
            ....|....|....|....|....|....|....|....|....|....|....|....|....|....|....|....|....|....|....|....|
BAC-51      GCAAACTGACACATTCCCGAAGCCAGGAAGAGCATATGTTTTTTTTATAATCACTAAGGGGCCTTATAGTGGCCACTAACAGTAACACTTGTATAAAAAA
BAC-51-15   GCAAACTGACACATTCCCGAAGCCAGGAAGAGCATATGTTTTTTTTATAATCACTAAGGGGCCTTATAGTGGCCACTAACAGTAACACTTGTATAAAAAA

                    157410    157420    157430    157440    157450    157460    157470    157480    157490    157500
            ....|....|....|....|....|....|....|....|....|....|....|....|....|....|....|....|....|....|....|....|
BAC-51      ACACACTCATTCACAGTGTGAACACACAGTTCACACTGTGAACGGAGCCTTTTCACAGTGTGGACACTGTCACAATGTGGTAAA--CCACACAGTATTCA
BAC-51-15   ACACACTCATTCACAGTGTGAACACACAGTTCACACTGTGAACGGAGCCTTTTCACAGTGTGGACACTGTCACAATGTGGTAAAAACCACACAGTATTCA

                    157510    157520    157530    157540    157550    157560    157570    157580    157590    157600
            ....|....|....|....|....|....|....|....|....|....|....|....|....|....|....|....|....|....|....|....|
BAC-51      CATTGTAAAATTCGATTTCACAGTGTGAATGTACCTGCTCACAGTGTGAAAATGATTTCACAGTGTGACCAAACTGTGATAAATAGATTTTCACAGTGTG
BAC-51-15   CATTGTAAAATTCGATTTCACAGTGTGAATGTACCTGCTCACAGTGTGAAAATGATTTCACAGTGTG---------------------------------
```

**Deletion 2, 5' end, this is likely an assembly artifact**

```
                    157610    157620    157630    157640    157650    157660    157670    157680    157690    157700
            ....|....|....|....|....|....|....|....|....|....|....|....|....|....|....|....|....|....|....|....|
BAC-51      AATACCTTACGACATACATTTCACGGTGTGAACAGTAAATTTCTCAGTGTGGCCACTCTGTGAAAAATACTTCACATTGTGAAAAAATAGAATTCACAGT
BAC-51-15   ----------------------------------------------------------------------------------------------------
```

```
                    157710    157720    157730    157740    157750    157760    157770    157780    157790    157800
                ....|....|....|....|....|....|....|....|....|....|....|....|....|....|....|....|....|....|....|....|
BAC-51          ATGGCCGCTGGAATACTAGTGTTTTGGATCGAGGCCAATCAAGTTTCCGTGATAACAGACGAATATGTACAACTACATGTTGACGGATGAGAACATAATT
BAC-51-15       ----------------------------------------------------------------------------------------------------

                    157810    157820    157830    157840    157850    157860    157870    157880    157890    157900
                ....|....|....|....|....|....|....|....|....|....|....|....|....|....|....|....|....|....|....|....|
BAC-51          GGAATGGCGGACATGGCCTGGTGAAGTTTCCCAACGGCTAGGATAGCTATCAGGCGAGGGTGTTGCCGGGCTGCTGCCGGGCTGTCATTGAATGGTGAGT
BAC-51-15       ----------------------------------------------------------------------------------------------------
```

**Sequence (2,270 bp) in BAC-51 that deleted from BAC-51-15 is omitted, this is likely an assembly artifact**

```
                    159510    159520    159530    159540    159550    159560    159570    159580    159590    159600
                ....|....|....|....|....|....|....|....|....|....|....|....|....|....|....|....|....|....|....|....|
BAC-51          TCCTGGAGTATGCGTGCGTGCACAGTGCGGGATCCTCACACCCAGGACAACATACACAGGCTGGAAATGGTACAGCTCCGCAACGCCCAGTTTATCACTG
BAC-51-15       ----------------------------------------------------------------------------------------------------

                    159610    159620    159630    159640    159650    159660    159670    159680    159690    159700
                ....|....|....|....|....|....|....|....|....|....|....|....|....|....|....|....|....|....|....|....|
BAC-51          GAGATCACCATCATACCAGCAGCATCGCACCAATGCTGCAACATCTCAAATGGCCATCTCTCCGAGAACGCAGGGCACGGTTTAAGATGGTGATAGTGTA
BAC-51-15       ----------------------------------------------------------------------------------------------------

                    159710    159720    159730    159740    159750    159760    159770    159780    159790    159800
                ....|....|....|....|....|....|....|....|....|....|....|....|....|....|....|....|....|....|....|....|
BAC-51          CGGAACTGTCAACCTGCACTCAGAGTTTCTCAAAATGTACCTTCTATCGGTGTTAACATCCTACCATGGTCATACCCAGTAATTCCAAATACAATTTACA
BAC-51-15       ----------------------------------------------------------------------------------------------------

                    159810    159820    159830    159840    159850    159860    159870    159880    159890    159900
                ....|....|....|....|....|....|....|....|....|....|....|....|....|....|....|....|....|....|....|....|
BAC-51          ATGACTCTCGTATACCAGAAGTCATTCTTCCCCGATTCCATCATGCTTTGGAACTTGCTACCTGCTGGATTTGTCAACTGTACTTTCGCTAAAGCTTTTA
BAC-51-15       --------------------------------------CATCATGCTTTGGAACTTGCTACCTGCTGGATTTGTCAACTGTACTTTCGCTAAAGCTTTTA
```

**Deletion 2, 3' end, this is likely an assembly artifact**

```
                    159910    159920    159930    159940    159950    159960    159970    159980    159990    160000
                ....|....|....|....|....|....|....|....|....|....|....|....|....|....|....|....|....|....|....|....|
BAC-51          AGCAAGAGGTACAAAATTTCCATCTCCGTTAGAAGGGGAAGAAGGATTTTTAACTGCTCATAGAATATTATAGAAAGTTTTAAATTCGCACTGTATATTC
BAC-51-15       AGCAAGAGGTACAAAATTTCCATCTCCGTTAGAAGGGGAAGAAGGATTTTTAACTGCTCATAGAATATTATAGAAAGTTTTAAATTCGCACTGTATATTC

                    160010    160020    160030    160040    160050    160060    160070    160080    160090    160100
                ....|....|....|....|....|....|....|....|....|....|....|....|....|....|....|....|....|....|....|....|
BAC-51          GCGACACCAGACCTGACGTACGTGATACACCATACGGTGGACTGGAGTGATATCTCAAGATCTGGGGGATGATAATTCGGGCTTTAACAAATTGTGTGAT
BAC-51-15       GCGACACCAGACCTGACGTACGTGATACACCATACGGTGGACTGGAGTGATATCTCAAGATCTGGGGGATGATAATTCGGGCTTTAACAAATTGTGTGAT

                    160110    160120    160130    160140    160150    160160    160170    160180    160190    160200
                ....|....|....|....|....|....|....|....|....|....|....|....|....|....|....|....|....|....|....|....|
BAC-51          AGTGGAGGTATTATTTTTGACTGCACAGTCGGAAGTTCCATCTAGTTATAATATCCTAACATCAGTATATTATCGCTAGAACAAAAAATCATAAGTTACT
BAC-51-15       AGTGGAGGTATTATTTTTGACTGCACAGTCGGAAGTTCCATCTAGTTATAATATCCTAACATCAGTATATTATCGCTAGAACAAAAAATCATAAGTTACT
```

```
                     160210    160220    160230    160240    160250    160260    160270    160280    160290    160300
                ....|....|....|....|....|....|....|....|....|....|....|....|....|....|....|....|....|....|....|....|
BAC-51          TATGTAGTTTAGGAAGTACATATATTTTTGTATGCCATTCTGCTTTTTTCTGTGCCACCGTATATAGAGTGTATGGTTATTTATTTGTAAAAAGATGAGC
BAC-51-15       TATGTAGTTTAGGAAGTACATATATTTTTGTATGCCATTCTGCTTTTTTCTGTGCCACCGTATATAGAGTGTATGGTTATTTATTTGTAAAAAGATGAGC

                     160310    160320    160330    160340    160350    160360    160370    160380    160390    160400
                ....|....|....|....|....|....|....|....|....|....|....|....|....|....|....|....|....|....|....|
BAC-51          AAAATCTGTTTTTTTTCTCATCTTCAGGTATTTATTAGTATTAGTTGTAGAAATTTATGTAGCCATAACAGTAAAACGTGTCTGCCTAAAATAGATTTGT
BAC-51-15       AAAATCTGTTTTTTTTCTCATCTTCAGGTATTTATTAGTATTAGTTGTAGAAATTTATGTAGCCATAACAGTAAAACGTGTCTGCCTAAAATAGATTTGT

                     160410    160420    160430    160440    160450    160460    160470    160480    160490    160500
                ....|....|....|....|....|....|....|....|....|....|....|....|....|....|....|....|....|....|....|
BAC-51          TTTCAACTTTTTTTCAAGATGTATTGACAGAGATATTTTGAAACTTTTTATCATTTTAATAGGTTTATGAAGAACCTGTATGTGCCAAAGATTTTCTGAT
BAC-51-15       TTTCAACTTTTTTTCAAGATGTATTGACAGAGATATTTTGAAACTTTTTATCATTTTAATAGGTTTATGAAGAACCTGTATGTGCCAAAGATTTTCTGAT

                     160510    160520    160530    160540    160550    160560    160570    160580    160590    160600
                ....|....|....|....|....|....|....|....|....|....|....|....|....|....|....|....|....|....|....|
BAC-51          ACATTGCAATTGCCAAAGTTACATGTCATGTGTGGATATTCTATACTTGGAAAATGTGACTAGCCATACTAATGTATCTTCATAAATTTAATATACCTAC
BAC-51-15       ACATTGCAATTGCCAAAGTTACATGTCATGTGTGGATATTCTATACTTGGAAAATGTGACTAGCCATACTAATGTATCTTCATAAATTTAATATACCTAC

                     160610    160620    160630    160640    160650    160660    160670    160680    160690    160700
                ....|....|....|....|....|....|....|....|....|....|....|....|....|....|....|....|....|....|....|
BAC-51          AGAAATGTTTAAGAGATATTGCCAGAGATGCTCTAAAACTATTTT-ATTACTGACGTAACATGCTAATGTGCTTTTATTATGTTTTACGAGTGTCAGTAC
BAC-51-15       AGAAATGTTTAAGAGATATTGCCAGAGATGCTCTAAAACTATTTTTATTACTGACGTAACATGCTAATGTGCTTTTATTATGTTTTACGAGTGTCAGTAC

                     160710    160720    160730    160740    160750    160760    160770    160780    160790    160800
                ....|....|....|....|....|....|....|....|....|....|....|....|....|....|....|....|....|....|....|
BAC-51          GAGTTCAGGGAAATATTGATGATATGTGCAATTTTTGCCGTCACTGTAATTTATTCCTCGATGTTATACAGTGGAACCTCTATAACTCTATTTCACAAGT
BAC-51-15       GAGTTCAGGGAAATATTGATGATATGTGCAATTTTTGCCGTCACTGTAATTTATTCCTCGATGTTATACAGTGGAACCTCTATAACTCTATTTCACAAGT

                     160810    160820    160830    160840    160850    160860    160870    160880    160890    160900
                ....|....|....|....|....|....|....|....|....|....|....|....|....|....|....|....|....|....|....|
BAC-51          CCACGTTTTTCTGTAATTCATTGTTTTAAACCCTTGTACAATAAGATATCTGATATAAAGAGGTCAATTTAGTGGACACCAATGTGCTCATTATAATGAG
BAC-51-15       CCACGTTTTTCTGTAATTCATTGTTTTAAACCCTTGTACAATAAGATATCTGATATAAAGAGGTCAATTTAGTGGACACCAATGTGCTCATTATAATGAG

                     160910    160920    160930    160940    160950    160960    160970    160980    160990    161000
                ....|....|....|....|....|....|....|....|....|....|....|....|....|....|....|....|....|....|....|
BAC-51          GTTTCACTGTAGATAGCAAGTTCTGTTTTCAGAAGTATCTCTACTATCTAATTTAAACTGTTTGGATTCATTTGTCTTTGTTTTCATTCTGGTTCACATC
BAC-51-15       GTTTCACTGTAGATAGCAAGTTCTGTTTTCAGAAGTATCTCTACTATCTAATTTAAACTGTTTGGATTCATTTGTCTTTGTTTTCATTCTGGTTCACATC

                     161010    161020    161030    161040    161050    161060    161070    161080    161090    161100
                ....|....|....|....|....|....|....|....|....|....|....|....|....|....|....|....|....|....|....|
BAC-51          ATGTTGGCATTCACAGAGTAAAATTTGTTTATCTGTAAGTATAGTATTACTGCAATCTTTGTATACCTAAGAGGTATATGTTCATAAGTCCTTTTAGCGA
BAC-51-15       ATGTTGGCATTCACAGAGTAAAATTTGTTTATCTGTAAGTATAGTATTACTGCAATCTTTGTATACCTAAGAGGTATATGTTCATAAGTCCTTTTAGCGA

                     161110    161120    161130    161140    161150    161160    161170    161180    161190    161200
                ....|....|....|....|....|....|....|....|....|....|....|....|....|....|....|....|....|....|....|
BAC-51          AAAGTACATCAATATAAGCTTTTGAAATACATTTTAAGTTACCTCATAAAGGCTATAACATGTATATGTTTAAGATTACTGTATTATTTTCAGATGTTTC
BAC-51-15       AAAGTACATCAATATAAGCTTTTGAAATACATTTTAAGTTACCTCATAAAGGCTATAACATGTATATGTTTAAGATTACTGTATTATTTTCAGATGTTTC
```

```
                 161210    161220    161230    161240    161250    161260    161270    161280    161290    161300
            ....|....|....|....|....|....|....|....|....|....|....|....|....|....|....|....|....|....|....|....|
BAC-51      GGGGGTATATTTCTATTTAAGTTGAGTTTATAAGTGAGACATTGATTATAAACCTTTTCTCCTTCGTATTTATACGTACTAAGCTCTACATGACTACATT
BAC-51-15   GGGGGTATATTTCTATTTAAGTTGAGTTTATAAGTGAGACATTGATTATAAACCTTTTCTCCTTCGTATTTATACGTACTAAGCTCTACATGACTACATT

                 161310    161320    161330    161340    161350    161360    161370    161380    161390    161400
            ....|....|....|....|....|....|....|....|....|....|....|....|....|....|....|....|....|....|....|....|
BAC-51      TTCATAGTTTGAAATATTTCACACAGAATTCAAAAACTAAACTTGTATCACAGTGTGGTGGGCGTTTCACAGTGTGGTAATCTCATACAATATAATGTGT
BAC-51-15   TTCATAGTTTGAAATATTTCACACAGAATTCAAAAACTAAACTTGTATCACAGTGTGGTGGGCGTTTCACAGTGTGGTAATCTCATACAATATAATGTGT

                 161410    161420    161430    161440    161450    161460    161470    161480    161490    161500
            ....|....|....|....|....|....|....|....|....|....|....|....|....|....|....|....|....|....|....|....|
BAC-51      CACAATGTAACGTAGTGGGCGTTTCACAGTGTGTTTGGTGTTTCACAGTGTGGTGGGCATTTCACTGTGTGAGTGTTCACAATATTAAATTGGGGCGTTT
BAC-51-15   CACAATGTAACGTAGTGGGCGTTTCACAGTGTGTTTGGTGTTTCACAGTGTGGTGGGCATTTCACTGTGTGAGTGTTCACAATATTAAATTGGGGCGTTT

                 161510    161520    161530    161540    161550    161560    161570    161580    161590    161600
            ....|....|....|....|....|....|....|....|....|....|....|....|....|....|....|....|....|....|....|....|
BAC-51      TCACATTGTGGTTGCTTCGTTTTACAGTGTGGTTCACAGTGTGGATATCTACACTGAAACGACGCGACCACAATGTGAAAATACCCCAATGTAACATTGT
BAC-51-15   TCACATTGTGGTTGCTTCGTTTTACAGTGTGGTTCACAGTGTGGATATCTACACTGAAACGACGCGACCACAATGTGAAAATACCCCAATGTAACATTGT

                 161610    161620    161630    161640    161650    161660    161670    161680    161690    161700
            ....|....|....|....|....|....|....|....|....|....|....|....|....|....|....|....|....|....|....|....|
BAC-51      GGGCGTTTCACAGTGTGTTTGCTGTTTCAAAGTGTGCTGGGCATTTCACTGTGTGAGTGTTTACAATGTTAAATTGGGGCGTTTCACATTGTGGTCGCTT
BAC-51-15   GGGCGTTTCACAGTGTGTTTGCTGTTTCAAAGTGTGCTGGGCATTTCACTGTGTGAGTGTTTACAATGTTAAATTGGGGCGTTTCACATTGTGGTCGCTT

                 161710    161720    161730    161740    161750    161760    161770    161780    161790    161800
            ....|....|....|....|....|....|....|....|....|....|....|....|....|....|....|....|....|....|....|....|
BAC-51      CGTTTCACAGTTGGGTTCACAGTATGAAACGACGCGACCACAATGTGAAAACGCCCCAATTTAACATTGTGGGCGTTACACAGTGTGGTGGGCATTTCAC
BAC-51-15   CGTTTCACAGTTGGGTTCACAGTATGAAACGACGCGACCACAATGTGAAAACGCCCCAATTTAACATTGTGGGCGTTACACAGTGTGGTGGGCATTTCAC

                 161810    161820    161830    161840    161850    161860    161870    161880    161890    161900
            ....|....|....|....|....|....|....|....|....|....|....|....|....|....|....|....|....|....|....|....|
BAC-51      TGTGTGAGTGTTCACAATGTTAAATTGGGGCGTTTTCACAGTGTGATTCACACTGTGTTTCACACTGTGAATGAGTGTTGTGGTCTGGTCTGGTCGCGAC
BAC-51-15   TGTGTGAGTGTTCACAATGTTAAATTGGGGCGTTTTCACAGTGTGATTCACACTGTGTTTCACACTGTGAATGAGTGTTGTGGTCTGGTCTGGTCGCGAC
```

**Alignment 2**. BAC-52 and BAC-52-2b

```
                   9910       9920       9930       9940       9950       9960       9970       9980       9990      10000
           ....|....| ....|....| ....|....| ....|....| ....|....| ....|....| ....|....| ....|....| ....|....| ....|....|
BAC52      CATACTGAAT ACTAAAAATT GTAAAATAAA -GATGTAGCT GAACTCATAC GAAGGTAATG TGCAATAATA AAGCCCCGAA TATTTTCCTG ACGCAGATGA
BAC52-2b   CATACTGAAT ACTAAAAAAT TGTAAAATAA AGATGTAGCT GAACTCATAC GAAGGTAATG TGCAATAATA AAGCCCCGAA TATTTTCCTG ACGCAGATGA

                  10010      10020      10030      10040      10050      10060      10070      10080      10090      10100
           ....|....| ....|....| ....|....| ....|....| ....|....| ....|....| ....|....| ....|....| ....|....| ....|....|
BAC52      ATTGATCATT GTTATTGAAT CAATGAGGAA GACGAAATGA AGGGAAAAGG AATACAATGT ATATAGAGAT AGAGAGAGAG AGAGAGAGGG --ATAGAGAG
BAC52-2b   ATTGATCATT GTTATTGAAT CAATGAGGAA GACGAAATGA AGGGAAAAGG AATACAATGT ATATAGAGAT AGAGAGAGAG AGAGAGAGAG GGATAGAGAG
```

```
                  10110      10120      10130      10140      10150      10160      10170      10180      10190      10200
           ....|....| ....|....| ....|....| ....|....| ....|....| ....|....| ....|....| ....|....| ....|....| ....|....|
BAC52      GGATATATAT ATATATATAT ATATATATAG AGAGAGAGAG AGAGAGAGAG AGA--GAATG AGATAGAGAG AAATAAAAGA GAGAGAGAGA GAA-GAAAGA
BAC52-2b   GGATATATAT ATATATATAT ATATATATAT AGAGAGAGAG AGAGAGAGAG AGA--GAATG AGATAGAGAG AAATAAAAGA GAGAGAGAGA GAAAGAAAGA

                  10210      10220      10230      10240      10250      10260      10270      10280      10290      10300
           ....|....| ....|....| ....|....| ....|....| ....|....| ....|....| ....|....| ....|....| ....|....| ....|....|
BAC52      GAGAGG-AGA GGTGAGATAG ATAGAAAGGG TAGAGAGAGA TGAAGAGACA AAAGAAATAG AGATATGTAG AGATAGAGAG AGCGAGAGGG AGAGACAAAG
BAC52-2b   GAGAGGGAGA GGTGAGATAG ATAGAAAGGG TAGAGAGAGA TGAAGAGACA AAAGAAATAG AGATATGTAG AGATAGAGAG AG--AGAGGG AGAGACAA-G

                  10310      10320      10330      10340      10350      10360      10370      10380      10390      10400
           ....|....| ....|....| ....|....| ....|....| ....|....| ....|....| ....|....| ....|....| ....|....| ....|....|
BAC52      AGACAGAGAG ACAAAGAGAA TATGAGAGTG AGATGGAGAG AGATATGAGT GTGAGAGAAA GAGG-AGAGA GAGAGAGAGA GAGAGAGAGA GAGAAGATAG
BAC52-2b   AGACAGAGAG ACAAAGAGAA TATGAGAGTG AGATGGAGAG AGATATGAGT GTGAGAGAAA GAGGGAGAGA GAGAGAGAGA GAGAGAGAGA GAGAAGATAG

                  10410      10420      10430      10440      10450      10460      10470      10480      10490      10500
           ....|....| ....|....| ....|....| ....|....| ....|....| ....|....| ....|....| ....|....| ....|....| ....|....|
BAC52      ATAAGATTAT TTATATATAC AATGCGTATA AAAAAAAGTG TGCCCAACTT TAGTAACCTC TACTTAAAAA TTTATAACAT ATAAACTGAT ATCCTGTCTA
BAC52-2b   ATAAGATTAT TTATATATAC AATGCGTATA AAAAAAAGTG TGCCCA-CTT TAGTAACCTC TACTTAAAAA TTTATAACAT ATAAACTGAT ATCCTGTCTA

                  10510      10520      10530      10540      10550      10560      10570      10580      10590      10600
           ....|....| ....|....| ....|....| ....|....| ....|....| ....|....| ....|....| ....|....| ....|....| ....|....|
BAC52      ATGATTTTAT ATTCATAATT GTACTGCCAT GGATGATTGA GCAGCAGGCT TTTTATAAAG TTTTTTCAAA ATCCTTTTTG AACCAAGTTT GGGCCGAAGC
BAC52-2b   ATGATTTTAT ATTCATAATT GTACTGCCAT GGATGATTGA GCAGCAGGCT TTTTATAAAG TTTTTTCAAA ATCCTTTTTG AACCAAGTTT GGGCCGAAGC

                  10610      10620      10630      10640      10650      10660      10670      10680      10690      10700
           ....|....| ....|....| ....|....| ....|....| ....|....| ....|....| ....|....| ....|....| ....|....| ....|....|
BAC52      AATGGATGCG GGTCAAAAGT CATTATGTGT GGGTTATCTC ATTCCATAAA CAATTGTTCT CTTATGAATA CTAACTATTA GGCTGTTGAA GATAAAAGGT
BAC52-2b   AATGGATGCG GGTCAAAAGT CATTATGTGT GGGTTATCTC ATTCCATAAA CAATTGTTCT CTTATGAATA CTAACTATTA GGCTGTTGAA GATAAAAGGT

                  10710      10720      10730      10740      10750      10760      10770      10780      10790      10800
           ....|....| ....|....| ....|....| ....|....| ....|....| ....|....| ....|....| ....|....| ....|....| ....|....|
BAC52      GTGAATATCA ACTCAAGCTA TAAAGGAAAT TTTATCACAA ACAAAATGTA TGATATTCTC TTTGTTATGA TGAATAAAAT CTTAAATTCA TTCGTATCCC
BAC52-2b   GTGAATATCA ACTCAAGCTA TAAAGGAAAT TTTATCACAA ACAAAATGTA TGATATTCTC TTTGTTATGA TGAATAAAAT CTTAAATTCA TTCGTATCCC

                  10810      10820      10830      10840      10850      10860      10870      10880      10890      10900
           ....|....| ....|....| ....|....| ....|....| ....|....| ....|....| ....|....| ....|....| ....|....| ....|....|
BAC52      TTGTTTAAGG AAACCTTTCT CAGTGATAAA AAACCGATCG GGACGTTATC TTCTTTATTA AAGTCTCATC GTAATAATAA TATGAAAAAT TTATATAGCG
BAC52-2b   TTGTTTAAGG AAACCTTTCT CAGTGATAAA AA--CGATCG GGACGTTATC TTCTTTATTA AAGTCTCATC GTAATAATAA TATGAAAAAT TTATATAGCG

                  10910      10920      10930      10940      10950      10960      10970      10980      10990      11000
           ....|....| ....|....| ....|....| ....|....| ....|....| ....|....| ....|....| ....|....| ....|....| ....|....|
BAC52      CTTGTGACAA AAGTTTCAAA GCACTCGTGT GTTCGTTCCT GCATTTGGAT GTAGTAACCT TTGAGTTTTC ATTCGCTATT CAATATAAAC ACCATAATGT
BAC52-2b   CTTGTGACAA AAGTTTCAAA GCACTCGTGT GTTCGTTCCT GCATTTGGAT GTAGTAACCT TTGAGTTTTC ATTCGCTATT CAATATAAAC ACCATAATGT

                  11010      11020      11030      11040      11050      11060      11070      11080      11090      11100
           ....|....| ....|....| ....|....| ....|....| ....|....| ....|....| ....|....| ....|....| ....|....| ....|....|
BAC52      GCTCATTACG CGTGTGAAAT TGTGTAGAGG ACAAGTGAGC AGAGAGAAAT TAGATATAAT GAAACAAGAA GTTAGTTGGC TGTTCAAAAT AACTAAAAGT
BAC52-2b   GCTCATTACG CGTGTGAAAT TGTGTAGAGG ACAAGTGAGC AGAGAGAAAT TAGATATAAT GAAACAAGAA GTTAGTTGGC TGTTCAAAAT AACTAAAAGT
```

```
                11110      11120      11130      11140      11150      11160      11170      11180      11190      11200
           ....|....| ....|....| ....|....| ....|....| ....|....| ....|....| ....|....| ....|....| ....|....| ....|....|
BAC52      TTTCCCCGGG GTCGTAACAA GGTTCTGCGA AACAGGGGCG GATCTAGCCG GCGGCGAGGG TGGGGGGGGG GGGG----CA ATTTAAGAAA ATAGTTAGCG
BAC52-2b   TTTCCCCGGG GTCGTAACAA GGTTCTGCGA AACAGGGGCG GATCTAGCCG GCGGCGAGGG TGGGG-AGGG GGGGGGGGCA ATTTAAGAAA ATAGTTAGCG

                11210      11220      11230      11240      11250      11260      11270      11280      11290      11300
           ....|....| ....|....| ....|....| ....|....| ....|....| ....|....| ....|....| ....|....| ....|....| ....|....|
BAC52      CCGAATTAGC CGGCGAAAAA GCAATAGGGG GGG--TTAAT GAGCAATAAA TTGTTATTTC TTTTTTGCTC AACGGGGGGG GGGGTAGTGC ATGCGCGGAT
BAC52-2b   CCGAATTAGC CGGCGAAAAA GCAATAGGGG GGGGGTTAAT GAGCAATAAA TTGTTATTTC TTTTTTGCTC AACGGGGGGG GGGG------ ----------
                                                                                                Deletion 1, 5' end

                11310      11320      11330      11340      11350      11360      11370      11380      11390      11400
           ....|....| ....|....| ....|....| ....|....| ....|....| ....|....| ....|....| ....|....| ....|....| ....|....|
BAC52      CCAGGGGAGG CCCCCCGCCC CCCAAAAAAG TTTTTAATTT TTTGTTTTTT TAAATGGAGA CAGATAAAAA TTTAGTGCTC ACTACCACCC CCCCCCCCCC
BAC52-2b   ---------- ---------- ---------- ---------- ---------- ---------- ---------- ---------- ---------- ----------

                11410      11420      11430      11440      11450      11460      11470      11480      11490      11500
           ....|....| ....|....| ....|....| ....|....| ....|....| ....|....| ....|....| ....|....| ....|....| ....|....|
BAC52      CCCCCTACTG AGCAAAATTT ATCGGCCGGC ACGATTTTCG AATTTCACCA CGCTAAATTA AAAATTGATT TCAAATTTGG GTCTCCCCTA AGGAATCCTG
BAC52-2b   ---------- ---------- ---------- ---------- ---------- ---------- ---------- ---------- ---------- ----------

                11510      11520      11530      11540      11550      11560      11570      11580      11590      11600
           ....|....| ....|....| ....|....| ....|....| ....|....| ....|....| ....|....| ....|....| ....|....| ....|....|
BAC52      GACCCGCGCC AGTAGTAAGC ACTATTTTTA TTTACTGAAC CCCCCCCCTT GAGCAAAAAC AAAAAGAAGG TAAGATTGTG GATACTATCT TTGTTTAAAT
BAC52-2b   ---------- ---------- ---------- ---------- ---------- ---------- ---------- ---------- ---------- ----------

               119410     119420     119430     119440     119450     119460     119470     119480     119490     119500
           ....|....| ....|....| ....|....| ....|....| ....|....| ....|....| ....|....| ....|....| ....|....| ....|....|
BAC52      TGCCTGAAAA AAAAATCACA GTAGAAATAT CCCCTGAAAA GGATTGATCA TCGTTTTCGA AATATTACCG GAGGTAGCTG GAAATTTGTT GATAAATATT
BAC52-2b   ---------- ---------- ---------- ---------- ---------- ---------- ---------- ---------- ---------- ----------

               119510     119520     119530     119540     119550     119560     119570     119580     119590     119600
           ....|....| ....|....| ....|....| ....|....| ....|....| ....|....| ....|....| ....|....| ....|....| ....|....|
BAC52      CCATGCCACA TGTGTAGTTT AATATTAGCC TACCCAATTT CGTTTATGTG TGCACTAAAA AGTCATTTGT CAGCATTTTC AGACATTCTG ATTAAATTTC
BAC52-2b   ---------- ---------- ---------- ---------- ---------- ---------- ---------- ---------- ---------- ----------

               119610     119620     119630     119640     119650     119660     119670     119680     119690     119700
           ....|....| ....|....| ....|....| ....|....| ....|....| ....|....| ....|....| ....|....| ....|....| ....|....|
BAC52      TTGTAATTTT GATGAATTCA AACCTTACAC TTCTGTACTA AACAGGCGCG TACGCAGGAT TTTTTCAAGT GGGGGGGGGG GGGGGTTTAA CATTTTTAAA
BAC52-2b   ---------- ---------- ---------- ---------- ---------- ---------- ---------- ---------- --GGGTTTAA CATTTTTAAA
                                                                                 Deletion 1, 3' end

               119710     119720     119730     119740     119750     119760     119770     119780     119790     119800
           ....|....| ....|....| ....|....| ....|....| ....|....| ....|....| ....|....| ....|....| ....|....| ....|....|
BAC52      TCGGGCCGAA AATTTCGCAT CGACTCAGCC AGCCGCTGAA TAAGCGGGGG GGGGGGGGGG GG--AAGAGG ACACTTTTTC TTTTCTTTTT TTGGTCTCGA
BAC52-2b   TCGGGCCGAA AATTTCGCAT CGACTCAGCC AGCCGCTGAA TAAGCGGGGG GGGGGGGGGG GGGGAAGAGG ACACTTTTTC TTTTCTTTTT TTGGTCTCGA
```

```
                119810     119820     119830     119840     119850     119860     119870     119880     119890     119900
            ....|....| ....|....| ....|....| ....|....| ....|....| ....|....| ....|....| ....|....| ....|....| ....|....|
BAC52       AATTTGAAAA TTTGACATTT TGCTCTGTTG GGGGGGGGGG GG-TAAGGTC GCCTTTTTTA GGTCAGCCAT GGGAATAGTT TTTTT-ATTA TTATTTTTTT
BAC52-2b    AATTTGAAAA TTTGACATTT TGCTCTGTTG GGGGGGGGGG GGGTAAGGTC GCCTTTTTTA GGTCAGCCAT GGGAATAGTT TTTTT-ATTA TTATTTTTTT

                119910     119920     119930     119940     119950     119960     119970     119980     119990     120000
            ....|....| ....|....| ....|....| ....|....| ....|....| ....|....| ....|....| ....|....| ....|....| ....|....|
BAC52       TTT-ATTATT CAAAAAAACA AAAAACAAAA CAAAACAAAG GGGGGGGGGG TTGTTTTTTT AGGGGGGTTT ATACACATAA AAATACCAAA GGGGGGGGG-
BAC52-2b    TT--ATTATT CAAAAAAACA AAAAACAAAA CAAAACAAAG GGGGGGGGGG TTGTTTTTTT AGGGGGGTTT ATACACATAA AAATACCAAA GGGGGGGGGG

                120010     120020     120030     120040     120050     120060     120070     120080     120090     120100
            ....|....| ....|....| ....|....| ....|....| ....|....| ....|....| ....|....| ....|....| ....|....| ....|....|
BAC52       TTAACCCCTA AACACCCCCC CCCCCCC--T GCGTACGCGC CTGGTACTAC ATACATATTA AACCTTTCTA TGTAAATTTT AGCCCATTTC TTTCAAAACG
BAC52-2b    TTAACCCCCT AAACACCCCC CCCCCCCCCT GCGTACGCGC CTGGTACTAC ATACATATTA AACCTTTCTA TGTAAATTTT AGCCCATTTC TTTCAAAACG

                120110     120120     120130     120140     120150     120160     120170     120180     120190     120200
            ....|....| ....|....| ....|....| ....|....| ....|....| ....|....| ....|....| ....|....| ....|....| ....|....|
BAC52       TAATCACATG ACCAGTACTA GATTTATTTT GAAACATCCG TCTTGATGTT TATAATGATA GATGACGTTA TACGACCCTG TCTTATGGGG TACCTTGTCA
BAC52-2b    TAATCACATG ACCAGTACTA GATTTATTTT GAAACATCCG TCTTGATGTT TATAATGATA GATGACGTTA TACGACCCTG TCTTATGGGG TACCTTGTCA

                120210     120220     120230     120240     120250     120260     120270     120280     120290     120300
            ....|....| ....|....| ....|....| ....|....| ....|....| ....|....| ....|....| ....|....| ....|....| ....|....|
BAC52       TTCGTGCCAT TTTGGGGGGA GGGTGGAGAC CCGCAGACTT CCCCCTCTAG AACCACTACT GAATACCACT GAACGCAACT GAAGAATATG TTATCGTCAT
BAC52-2b    TTCGTGCCAT TTTGGGGGGA GGGTGGAGAC CCGCAGACTT CCCCCTCTAG AACCACTACT GAATACCACT GAACGCAACT GAAGAATATG TTATCGTCAT

                120310     120320     120330     120340     120350     120360     120370     120380     120390     120400
            ....|....| ....|....| ....|....| ....|....| ....|....| ....|....| ....|....| ....|....| ....|....| ....|....|
BAC52       AGAGGTCGGT TATACATTTT TTTCCATGCA GGTTTTCATT CACTGTGAGG TGCTCATTTG TAATGATAAT GACCCATCGT CTCGGTGTTC CCAGGGATGT
BAC52-2b    AGAGGTCGGT TATACATTTT TTTCCATGCA GGTTTTCATT CACTGTGAGG TGCTCATTTG TAATGATAAT GACCCATCGT CTCGGTGTTC CCAGGGATGT

                120410     120420     120430     120440     120450     120460     120470     120480     120490     120500
            ....|....| ....|....| ....|....| ....|....| ....|....| ....|....| ....|....| ....|....| ....|....| ....|....|
BAC52       AAGTCTAGAT TCAGACGTGG TAGCCGTCAC ACCCGTGGAG CAAGCTCCAC TCCTCATCTA ATCTCTAATG GACCTCTTTC AACTGCCCAC ACTATGCATG
BAC52-2b    AAGTCTAGAT TCAGACGTGG TAGCCGTCAC ACCCGTGGAG CAAGCTCCAC TCCTCATCTA ATCTCTAATG GACCTCTTTC AACTGCCCAC ACTATGCATG

                120510     120520     120530     120540     120550     120560     120570     120580     120590     120600
            ....|....| ....|....| ....|....| ....|....| ....|....| ....|....| ....|....| ....|....| ....|....| ....|....|
BAC52       CCGCAGAAGC ACACAACTCT GGTAATTACC TTTTTAAAGA ACACAACACT TAACTTTAAA GGTGTCGTGT TTGTCTAGTG AAAACGTCGT TGGACTGTGG
BAC52-2b    CCGCAGAAGC ACACAACTCT GGTAATTACC TTTTTAAAGA ACACAACACT TAACTTTAAA GGTGTCGTGT TTGTCTAGTG AAAACGTCGT TGGACTGTGG
```

## Reference

1. Oren M, Barela Hudgell MA, D' Allura B, Agronin J, Gross A, Podini D, et al. Short tandem repeats, segmental duplications, gene deletion, and genomic instability in a rapidly diversified immune gene family. BMC Genomics. 2016;17:900.
